# Analysis of the signal cross talk via CCL26 in the tumor microenvironment in osteosarcoma

**DOI:** 10.1038/s41598-021-97153-2

**Published:** 2021-09-13

**Authors:** Masanori Kawano, Tatsuya Iwasaki, Ichiro Itonaga, Yuta Kubota, Kazuhiro Tanaka, Hiroshi Tsumura

**Affiliations:** Department of Orthopaedic Surgery, Faculty of Medicine, Oita University, Oita, 879-5593 Japan

**Keywords:** Cancer, Oncology

## Abstract

Interaction with surrounding healthy cells plays a major role in the growth and metastasis of osteosarcoma. In this study, we hypothesized that humoral factors, which do not require direct contact with cells, are involved in the interaction between osteosarcoma and the surrounding cells. We identified the humoral factor involved in the association between tumor cells and surrounding normal cells using a co-culture model and investigated the significance of our findings. When human osteosarcoma cells (MG63) and human mesenchymal stem cells (hMSCs) were co-cultured and comprehensively analyzed for changes in each culture group, we found that the expression of chemokine (CC motif) ligand 26 (CCL26) was significantly enhanced. We also analyzed the changes in cell proliferation in co-culture, enhanced interaction with administration of recombinant CCL26 (rCCL26), reduced interaction with administration of anti-CCL26 antibodies, changes in invasive and metastatic abilities. CCL26 levels, motility, and invasive capability increased in the co-culture group and the group with added rCCL26, compared to the corresponding values in the MG63 single culture group. In the group with added CCL26 neutralizing antibodies, CCL26 level decreased in both the single and co-culture groups, and motility and invasive ability were also reduced. In a nude mice lung metastasis model, the number of lung metastases increased in the co-culture group and the group with added rCCL26, whereas the number of tumors were suppressed in the group with added neutralizing antibodies compared to those in the MG63 alone. This study identified a possible mechanism by which osteosarcoma cells altered the properties of normal cells to favorably change the microenvironment proximal to tumors and to promote distant metastasis.

## Introduction

Osteosarcoma is the most common primary malignant bone tumor, and advances in chemotherapy have improved survival rates, but there are still cases of distant metastasis, leading to poor prognosis. Recently, it has been established that the malignant trait of tumor cells does not only involve proliferative and metastatic activity but also the interaction between them and the normal cells surrounding them^1,2^. The existence of humoral factors that exert their effects even without direct contact between involved cells may be of significant importance for distant metastasis. Based on this perspective, we analyzed how osteosarcoma cells interact with different types of human mesenchymal stem cells (hMSCs). We have previously developed a co-culture system of osteosarcoma cells (MG63) and hMSCs under non-contact conditions^3^. Whole genome analysis of the mRNA altered by the co-culture system using cDNA arrays revealed that a chemokine, CCL26, was mutually released by cells.

CCL26 is a CCR3 ligand mainly expressed on the surface of eosinophil cells and plays an important role during cell migration and invasion to sites of inflammation^4–6^. However, it has also been reported that in malignant tumors, invasion and metastasis are promoted by signaling via CCL26 or CCR3^6–8^. Furthermore, CCR3, a receptor for CCL26, includes signaling factors such as Rac and Src and downstream factors which positively regulate malignant tumors^9–12^.

The progression of malignant tumor cells in a living body is not only affected by the cells themselves but also by the interaction with the normal cells surrounding the tumor cells. How do the properties of normal cells, which happen to surround tumor cells, change when the cells are in close proximity to malignant tumors? We used a co-culture model and discovered that the chemokine CCL26 might be involved in the progression of osteosarcoma cells. The purpose of the study was to elucidate the involvement and significance of CCL26 in the tumor microenvironment and distant metastasis in osteosarcoma.

## Results

### mRNA expression in co-cultured condition

**Figure 1:**
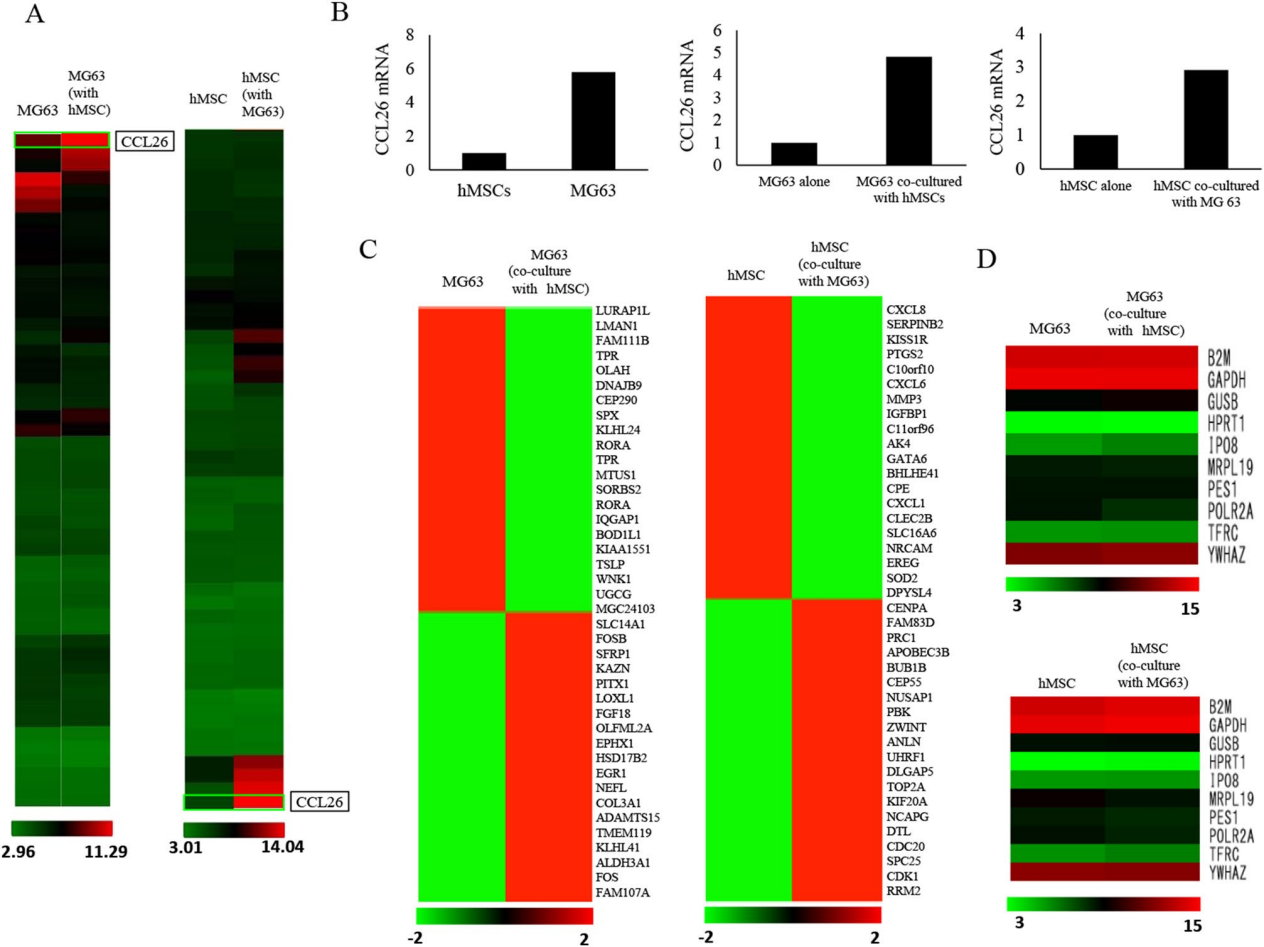
cDNA array profiling of genetic alterations and the expression of CCL26 in mono-cultured and co-cultured MG63 cells and hMSCs. (**A**) Changes in mRNA expression in hMSCs at 48 h after the co-culture with MG63 and in MG63 co-cultured with hMSCs. Co-culture condition increased the expression of CCL26 mRNA in both cell lines. (**B**) Relative expression of CCL26 mRNA in alone and co-cultured condition in each cell lines. Changes in the mRNA expression with high expression variability (**C**) and changes in the expression of housekeeping genes (control) (**D**).

We investigated mRNA expression in MG63 monoculture and MG63 cells co-cultured with hMSCs. Similarly, we also investigated mRNA expression in hMSC monoculture and hMSCs co-cultured with MG63 cells (Fig. 1A). As shown previously, high CCL26 expression was observed in MG63 cells in the mono-culture state, and the level of expression was about 5.81-fold higher than that observed in hMSCs. We observed that CCL26 mRNA expression increased by 4.82-fold in hMSCs cells co cultured with MG63 and CCL26 it increased by 2.92-fold in MG63 co-cultured with hMSCs (Fig. 1B). Data on the expression levels of the top 20 up-regulated genes and the top 20 down-regulated genes are shown (Fig. 1C). Changes in the expression of housekeeping genes are shown as a control (Fig. 1D). There was no remarkable change in the expression of the housekeeping genes, even under co-culture conditions.

### Effects of recombinant CCL26 on mRNA expression and cell proliferation

**Figure 2:**
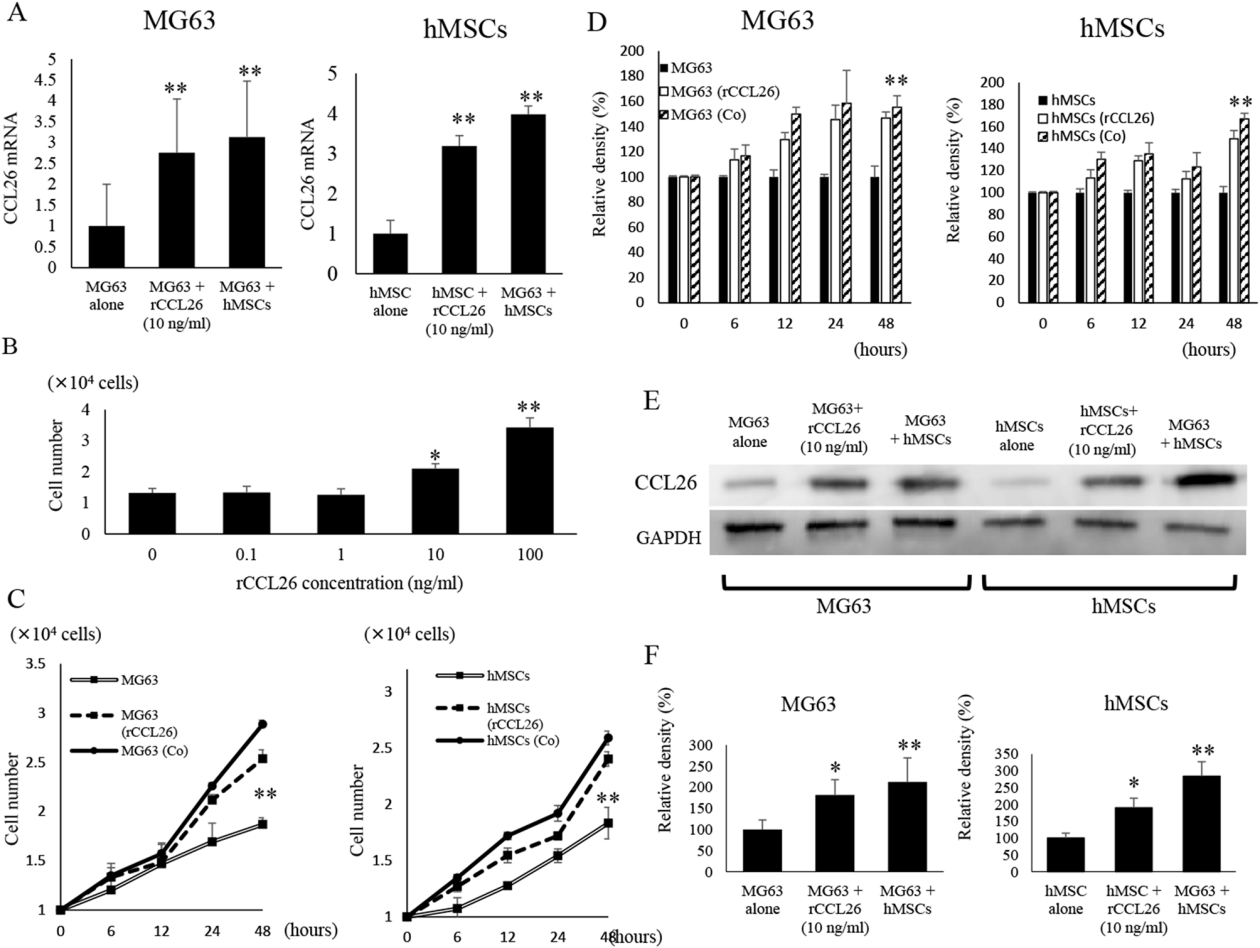
Changes in CCL26 expression and cell growth in MG63 and hMSCs induced by the co-culture condition and rCCL26 administration. (**A**) Changes in CCL26 mRNA expression in MG63 and hMSCs were assessed by qRT-PCR. The co-culture and rCCL26 addition significantly increased the expression of mRNA of CCL26. (**B**) rCCL26 was administered to mono-cultured MG63. There was a significant increase in cell growth with rCCL26 at 10 ng/ml. (*) p < 0.05, (**) p < 0.01. (**C**) Changes in cell proliferation induced by Co-cultured condition and recombinant CCL26 administration in MG63 and hMSCs. (**D**) Changes in the degree of cell proliferation in the untreated, co-culture, and recombinant groups were analyzed by performing cell proliferation assay via BrdU incorporation. (**E**) Changes inCCL26 protein expression of intra-cellular hMSCs and MG63 were assessed by western blot analysis. The co-culture and addition of rCCL26 increased the expression ofCCL26 protein in MG63 and hMSCs. (**F**) The quantification of western blot analysis. Data represents represent the mean ± SD of three independent experiments. p < 0.05 was considered to indicate significance: (*) p < 0.05, (**) p < 0.01.

An increase in CCL26 mRNA levels was observed after recombinant CCL26 (rCCL26) administration to MG63 and hMSC culture dishes. MG63 cells administered with rCCL26 (276 ± 43%) (p < 0.01) and co-cultured with hMSCs (313 ± 29%) (p < 0.01) showed higher levels of CCL26 mRNA expression compared to the expression observed in MG63 alone group (100%). hMSCs cells administered with rCCL26 (319 ± 26%) (p < 0.01) and co-cultured with MG63 (398 ± 21%) (p < 0.01) showed higher levels of CCL26 mRNA expression compared to the expression observed in hMSCs alone group (100%) (Fig. 2A). The effects of rCCL26 on cell proliferation were investigated in MG63 cells and hMSCs. rCCL26 at 10 ng/ml (2.1 ± 0.16 × 10^5^ cells) administration increased cell proliferation in MG63 cells as compared to 1 ng/ml (1.26 ± 0.19 × 10^5^ cells) as determined by cell growth assay (p < 0.05) (Fig. 2B). The cell growth of co-cultured MG63 (2.89 ± 0.4 × 10^5^ cells) and rCCL26 group (2.53 ± 0.1 × 10^5^ cells) were significantly increased compared with MG63 alone (1.97 ± 0.61 × 10^5^ cells) (p < 0.01). The cell growth of co-cultured hMSCs (2.59 ± 0.14 × 105 cells) and rCCL26 administered cells (2.4 ± 0.6 × 10^5^ cells) were significantly increased compared with hMSCs alone (1.83 ± 0.64 × 10^5^ cells) (p < 0.01) (Fig. 2C). Next, we used a bromodeoxyuridine (BrdU) proliferation assay to determine the effects of rCCL26 (146.7 ± 4.9%) and co-culture condition (155.4 ± 9.1%) compared to the proliferation of untreated MG63 cells (100%). The rCCL26 (148.9 ± 7.5%) and co-culture group (167 ± 5.1%) showed markedly increased proliferation than the untreated group using BrdU assays in hMSCs (100%). (Fig. 2D). Additionally, we observed that the expression of intracellular CCL26 proteins in rCCL-26 administration (182 ± 16.3%) (p < 0.05) and co-cultured with hMSCs (212 ± 28%) (p < 0.01) significantly increased compared to levels with MG63 alone. We performed immunoblot analysis to evaluate the protein levels of CCL26 in each group (Fig. 2E). Western blot analysis showed that the expression levels of CCL26 in MG63 were significantly increased with rCCL26 administration (182 ± 16.3%) (p < 0.05) and MG63 co-cultured with hMSCs (212 ± 28%) (p < 0.01) compared to levels with MG63 alone. CCL26 expression levels in hMSCs were significantly increased after rCCL26 administration to hMSCs (190 ± 28%) (p < 0.05) and hMSCs co-cultured with MG63 (284 ± 43%) (p < 0.01) (Fig. 2F).

### Inhibition of CCL26 expression by anti-CCL26 antibodies

**Figure 3:**
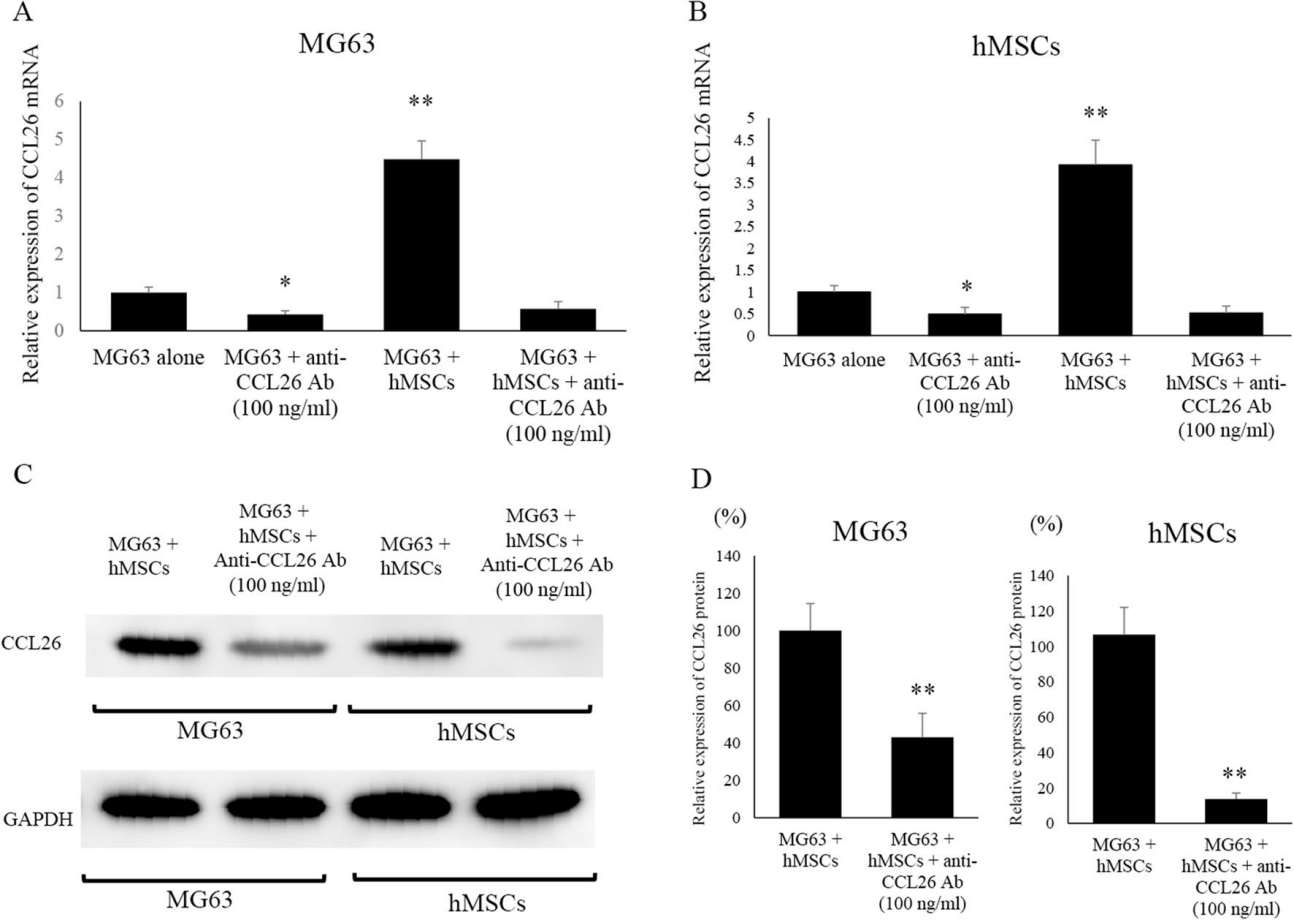
Effects of neutralizing anti-CCL26 Ab on CCL26 expression in mono-cultured and co-cultured MG63 and hMSCs. (**A**) Changes inCCL26 expression in MG63 were assessed by qRT-PCR. The addition of antiCCL26 Ab to MG63 decreased the expression ofCCL26 mRNA. (**B**) Changes inCCL26 expression in hMSCs were assessed by qRT-PCR. The addition of anti-CCL26 Ab to hMSCs decreased the expression ofCCL26 mRNA. (**C**) Changes inCCL26 protein expression in hMSCs and MG63 were assessed by western blot analysis. The addition of anti-CCL26 Ab to MG63 and hMSCs decreased the expression ofCCL26 protein in these cells. (**D**) The quantification of western blot analysis. Data represents represent the mean ± SD of three independent experiments. p < 0.05 was considered to indicate significance: (*) p < 0.05, (**) p < 0.01.

Blockade of the CCL26 communication loop using anti-CCL26 mAb resulted in reduced CCL26 levels in both MG63 and hMSC in co-culture. Indeed, MG63 co-cultured with hMSCs and treated with anti-CCL26 Ab showed lower CCL26 mRNA levels (58 ± 4.8%) (p < 0.05) than untreated MG63 co-cultured with hMSCs (449 ± 52.4%) (p < 0.01) (Fig. 3A). We observed decreased CCL26 mRNA levels (52 ± 15.7%) (p < 0.05) in hMSCs co-cultured with MG63 and treated with anti-CCL26 Ab compared to those in hMSCs co-cultured with MG63 (392 ± 56.5%) (p < 0.01) (Fig. 3B). Western blot analysis showed that CCL26 protein expression levels in co-cultured MG63 and hMSCs were dramatically decreased following with anti-CCL26 Ab administration (Fig. 3C). CCL26 expression levels in MG63 cells were significantly decreased after anti-CCL26 Abs administration to in MG63 co-cultured with hMSCs and treated with anti-CCL26 Ab (34 ± 1.7%) (p < 0.01) compared to levels with MG63 co-cultured with hMSCs (100%). The level of CCL26 protein expression in hMSCs after anti-CCL26 Abs administration to in hMSCs co-cultured with MG63 and treated with anti-CCL26 Ab (13.8 ± 3.3%) (p < 0.01) was down regulated compared to levels inwith hMSCs co-cultured with MG63 (100%) (Fig. 3D).

### Changes in expression of Rac and its related motility factors

**Figure 4:**
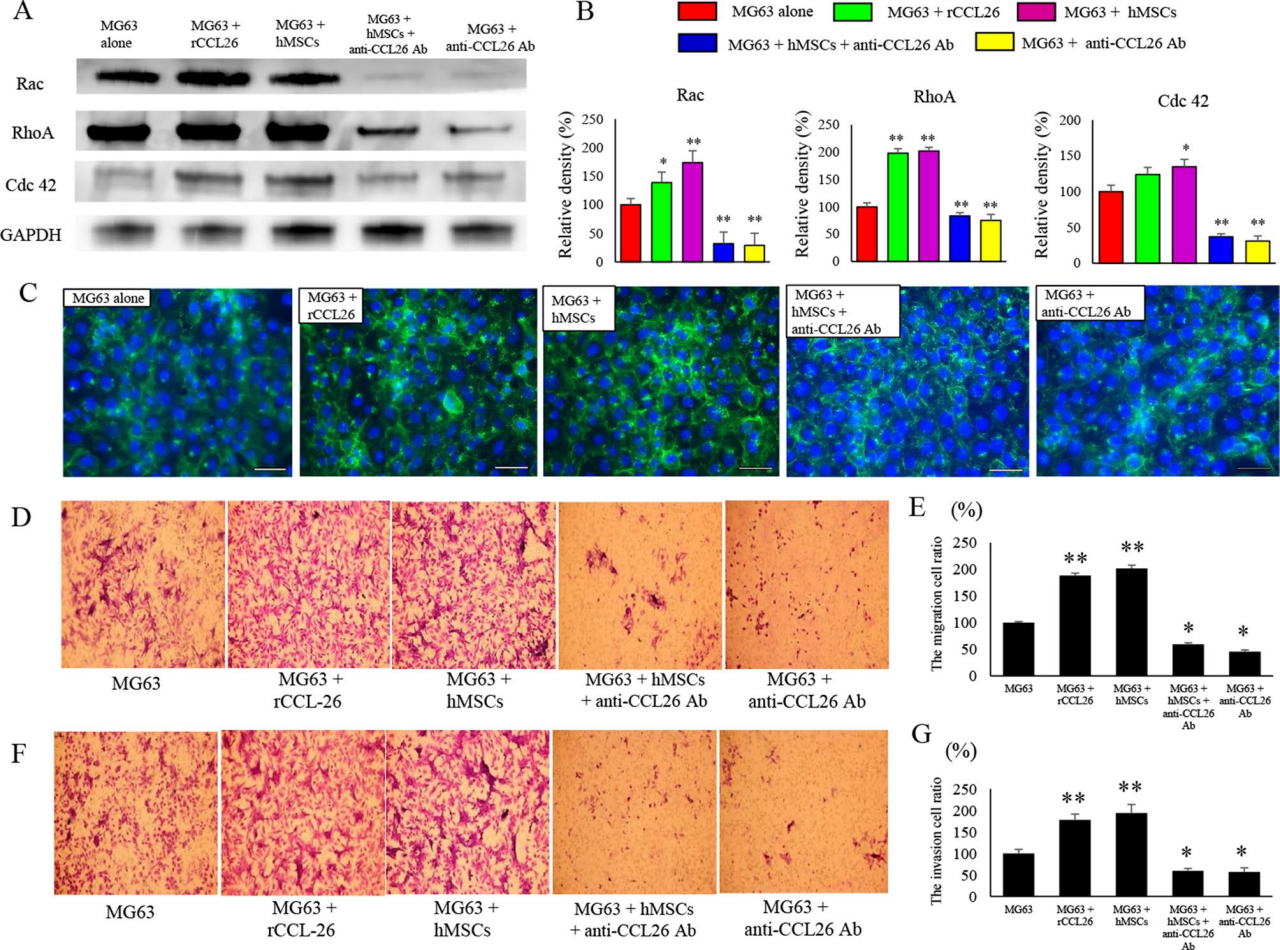
Effects of co-culture condition and administration of rCCL26 or anti-CCL26 Ab on motility of MG63. (**A**) Changes in protein expression of factors related to cell motility. (**B**) The quantification of western blot analysis. Data represents represent the mean ± SD of three independent experiments. (**C**) The influence of co-culture and rCCL26 or anti-CCL26 Ab on actin fiber morphology was evaluated using immunofluorescent imaging. Original magnification, × 400; Scale bars: 50 μm. (**D**) The cell migration of MG63 was assessed in each group at 24 h after the challenge with or without rCCL26 and neutralizing anti-CCL26 Ab. (**E**) The amount of MG63 cells that crossed the membrane was measured. A significant decrease in motility was found in the group given anti-CCL26 Ab. (**F**) The cell invasion in MG63 was assessed in each group after 24 h. (**G**) The amount of the cells of which the membrane with Matrigel was crossed by MG63 was measured. Decreased migration ability was found in the group administered anti-CCL26 Ab. p < 0.05 was considered to indicate significance: (*) p < 0.05, (**) p < 0.01.

Since Rac, RhoA, and Cdc42 are key molecules of cell motility^13–15^, we confirmed their protein expression levels in each group (Fig. 4A). Upon co-culture with hMSCs, the expression levels of Rac 1/2/3 (174.2 ± 20.5%), RhoA (202 ± 6.6%), and Cdc42 (135.6 ± 10.2%) in MG63 were increased compared to those in MG63 alone (100%) (p < 0.01). However, the expression levels of Rac 1/2/3 (32.7 ± 4.7%), RhoA (73 ± 5.7%), and Cdc42 (37.3 ± 4.2%) in MG63 were dramatically decreased following anti-CCL26 Ab administration compared with those in MG63-alone cells (p < 0.01). When MG63 cells were co-cultured with anti-CCL26 Ab, the expression levels of Rac 1/2/3 (29.6 ± 3.8%), RhoA (72.7 ± 3.3%), and Cdc42 (31.5 ± 7.5%) were also dramatically decreased in co-cultured cells (p < 0.01) (Fig. 4B).

### Anti-CCL26 antibody restricts extension of the actin fibers in MG63 cells

To evaluate the invasive ability of the tumor cells was morphologically changed was assessed by fluorescent staining of actin fibers. The actin fibers are extended in MG63 cells after pulsing with rCCL26 and MG63 co-cultured with hMSCs cells whereas the actin fibers are observed to be reduced in the cells to which MG63 co-cultured with hMSCs and with anti-CCL26 Ab (Fig. 4C).

### Intensification of cell motility of MG63 by co-culture and CCL26

To confirm the influence of CCL26 on cell migration (Fig. 4D), we performed a transwell motility assay using co-cultured MG63 and hMSCs with or without rCCL26 and anti-CCL26 Ab. MG63 cells after pulsing with rCCL26 (188.1 ± 4.1%) and MG63 after co-culturing with hMSCs (201.7 ± 6%) showed statistically increased motility capacity compared to that with MG63 alone (100%) (p < 0.01). MG63 with anti-CCL26 Ab (45.7 ± 3.4%) showed impaired migration ability in comparison with that of MG63 alone (p < 0.05) (Fig. 4E). In a matrigel invasion assay, anti-CCL26 Ab attenuated the invasiveness of MG63 and MG63 co-cultured with hMSCs (Fig. 4F). MG63 cells after pulsing with rCCL26 (178.1 ± 13.2%) and MG63 co-cultured with hMSCs (193.8 ± 20.3%) showed statistically increased migration capacity compared to that of MG63 alone (100%) (p < 0.01). Transwell cells that proceeded through the membrane with matrigel coated in MG63 with anti-CCL26 Ab (57.2 ± 9.5%) were significantly lower in comparison with those with MG63 alone (100%) (p < 0.05) (Fig. 4G).

### Changes in expression of Src and its downstream factors

**Figure 5:**
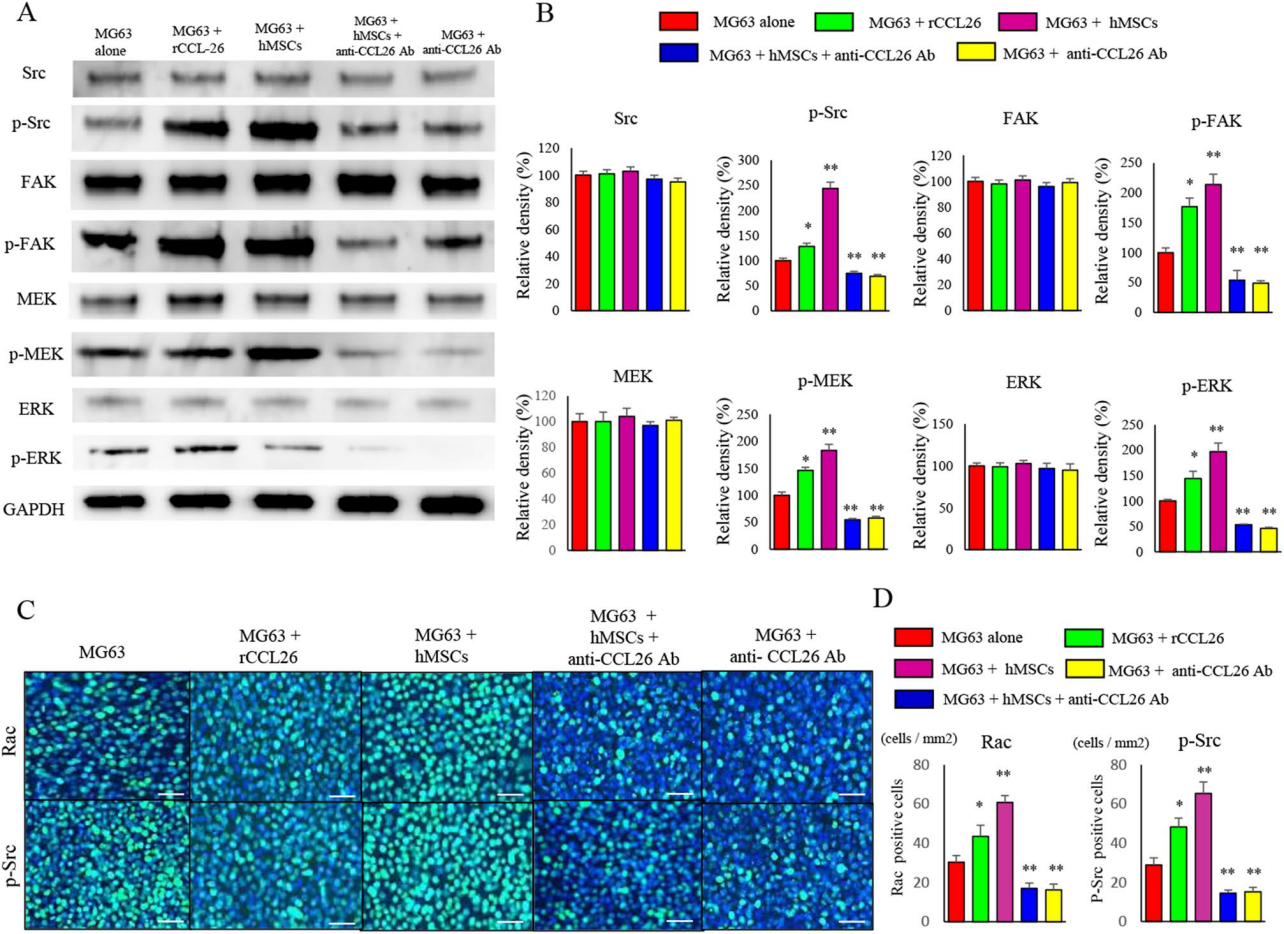
Changes in expression of Src and its downstream factors related to invasive potential. (**A**) Changes in phosphorylation and the expression of protein factors relating to invasive potential were analyzed. Decreased phosphorylation of Src, FAK, MEK and ERK in MG63 cells was noted in the group administered anti-CCL26 Ab. (**B**) The quantification of western blot analysis. (**C**) Immunofluorescence staining of cultured MG63 cells showed decreased CCL26 and p-Src in the group administered anti-CCL26 Ab. Original magnification, × 400; Scale bars: 50 μm. (**D**) The number of Rac and p-Src positive cells per unit area. Data represents represent the mean ± SD of three independent experiments. p < 0.05 was considered to indicate significance: (*) p < 0.05, (**) p < 0.01.

Since cell motility was remarkably enhanced in co-cultured MG63 with hMSCs, and Src is known to be a key regulatory molecule for cell motility, immunoblot analyses were carried out to investigate the expression of Src and its downstream factors (Fig. 5A). The levels of the protein expression of Src, FAK, MEK and ERK in MG63 were not significantly affected by co-culture or rCCL26 and anti-CCL26 administration. However, the protein expression levels of the phosphorylated (p) forms of Src (p-Src) (130.5 ± 5.1%) (p < 0.05), p-FAK (177 ± 14.2%) (p < 0.05), p-MEK (146.9 ± 5.8%) (p < 0.05), and p-ERK (144 ± 14.6%) (p < 0.05) in MG63 were elevated by the presence of rCCL26. The protein expression levels of p-Src (244.6 ± 16.7%) (p < 0.01), p-FAK (214.9 ± 17.8%) (p < 0.01), p-MEK (183.3 ± 10.9%) (p < 0.01), and p-ERK (197 ± 16.8%) (p < 0.01) in MG63 were elevated by the co-culture with hMSCs. In contrast, p-Src (68.8 ± 3.4%) (p < 0.01), p-FAK (49.6 ± 3.9%) (p < 0.01), p-MEK (57.9 ± 3.4%) (p < 0.01), and p-ERK (46.1 ± 8.3%) (p < 0.01) expression in MG63 were dramatically decreased with anti-CCL26 Ab administration compared with MG63 alone cells. Western blot analysis further demonstrated that co-cultured cells with anti-CCL26 Ab administration dramatically decreased the expression levels of p-Src (74.9 ± 3.7%) (p < 0.01), p-FAK (54.6 ± 6.3%) (p < 0.01), p-MEK (55.5 ± 5.1%) (p < 0.01), and p-ERK (53.8 ± 4.6%) (p < 0.01) in compared with MG63 alone (Fig. 5B).

### Analysis of factors involved in motility by performing fluorescence immunostaining

Immunofluorescence staining was used to evaluate Rac and p-Src expression in MG63 cells co-cultured under various conditions. Rac and p-SRC expression increased significantly in the MG63 cells administered with rCCL26, as well as the co-cultured groups. In contrast, Rac and p-Src expression significantly decreased in all neutralizing antibody-groups (Fig. 5C). The number of cells positive for Rac expression was significantly increased in treated with rCCL26 (43.5 ± 5.7 cells/mm^2^) (p < 0.05) or MG63 co-cultured with hMSCs cells (60.8 ± 3.5 cells/mm^2^) (p < 0.01) compared to MG63 alone (30.3 ± 3.4 cells/mm^2^). The number of cells positive for Rac expression was significantly decreased administered anti-CCL26 Ab with MG63 (16.2 ± 3 cells/mm^2^) (p < 0.01). Moreover, the number of cells positive for p-Src expression, which was significantly increased administered with rCCL26 (48.3 ± 4.5 cells/mm^2^) (p < 0.05) or MG63 co-cultured with hMSCs cells (65.5 ± 5.6 cells/mm^2^) (p < 0.01) compared to MG63 alone (28.8 ± 3.7 cells/mm^2^). However, the number of cells positive for p-Src expression was significantly decreased administered anti-CCL26 Ab with MG63 (15.2 ± 2.3 cells/mm^2^) (p < 0.01) compared to MG63 alone (p < 0.05) (Fig. 5D).

### Inhibition of tumor metastasis in a nude mice xenograft model by CCL26 suppression

**Figure 6:**
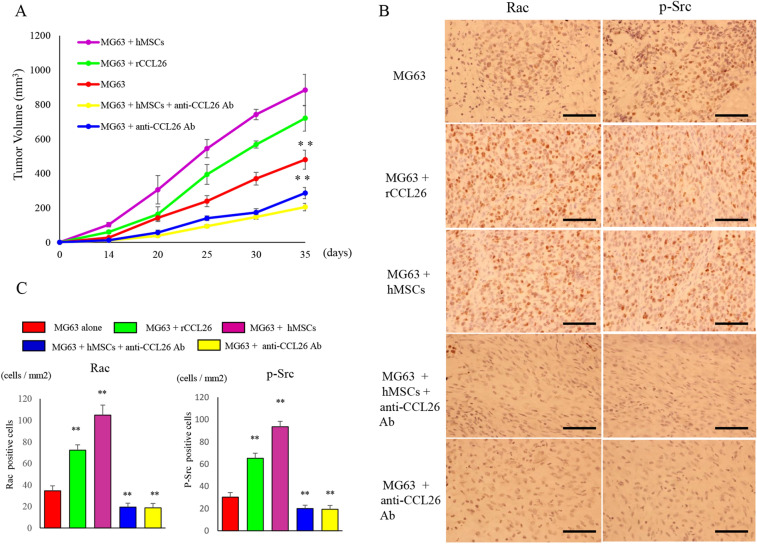
Changes in the lung nodules and the expression of Rac and phosphorylated Src in pulmonary metastatic lesions. (**A**) The group given anti-CCL26 Ab showed a significant suppression of the size of the pulmonary metastatic lesion. (**B**) Immunostaining of the tissues collected from the pulmonary metastatic lesion. Decreased expression of Rac and p-Src was observed in the group administered anti-CCL26 Ab. Original magnification, × 400; Scale bars: 50 μm. (**C**) The number of Rac and p-Src positive cells per unit area. Data represents represent the mean ± SD of three independent experiments. p < 0.05 was considered to indicate significance: (*) p < 0.05, (**) p < 0.01.

We next investigated the efficacy of CCL26 silencing against osteosarcoma tumor metastasis in vivo (Fig. 6A). The suppression of CCL26 by anti-CCL26 Ab in MG63 cells co-cultured with hMSCs resulted in a significant decrease in the growth of metastatic tumors in nude mice. MG63 cells after pulsing with rCCL26 (720.7 ± 74.9 mm^3^) and MG63 after co-culturing with hMSCs (884.3 ± 91.1 mm^3^) showed statistically larger lung tumors in mice compared to MG63 alone (480.2 ± 55.2 mm^3^) (p < 0.01). On the other hand, MG63 cells after administration of anti-CCL26 Ab (306.4 ± 21.8 mm^3^) showed statistically smaller lung tumors in mice compared to MG63 alone (p < 0.01). Immunohistochemistry analysis using resected lung tumors demonstrated that the expression of Rac and p-Src was reduced in the anti-CCL26 Ab-administered tumor tissues (Fig. 6B). The number of cells that tested positive for Rac expression was significantly increased in mice administered with rCCL26 (72.3 ± 4.9 cells/mm^2^) or MG63 co-cultured with hMSCs (104.8 ± 9.3 cells/mm^2^) compared to that with MG63 alone (34.5 ± 4.6 cells/mm^2^). In contrast, the number of cells that tested positive for Rac expression was significantly decreased in mice administered anti-CCL26 Ab with MG63 (18.7 ± 4.1 cells/mm^2^) (p < 0.01). The number of cells that tested positive for p-Src expression was significantly increased in mice administered with rCCL26 (65.2 ± 4.6 cells/mm^2^) or MG63 co-cultured with hMSCs (93.5 ± 4.9 cells/mm^2^) compared to that with MG63 alone (32.7 ± 4.4 cells/mm^2^). In contrast, in mice administered anti-CCL26 Ab with MG63 (19.1 ± 3.6 cells/mm^2^), the number of cells that tested positive for p-Src expression was significantly decreased compared to that with MG63 alone (p < 0.05). (Fig. 6C).

## Discussion

Standard treatment of osteosarcoma consists mainly of neoadjuvant chemotherapy^16,17^, and treatment results have gradually improved. However, in many cases, the tumors are resistant to treatment, and the prognosis of metastasizing tumors is extremely poor. Metastasis is the most important event that directly affects the prognosis of osteosarcoma, and thus controlling it is the key to improving the outcome of osteosarcoma treatment. During metastasis, osteosarcoma cells have to survive in an environment completely different from that of the primary tumor, and interaction with surrounding normal cells is very important for these tumor cells. Osteosarcoma cells are not supposed to survive under conditions in which they are surrounded by normal cells unless osteosarcoma cells establish an environment that is convenient for their survival. Thus, it is very likely that the surrounding normal cells are somehow exploited by the tumor cells. Regarding the interaction between normal and tumor cells, we assumed that it would be more efficient for the cells to cross talk via a humoral factor that does not require cell–cell contact. To test this hypothesis, we have established a co-culture system in which osteosarcoma cells and mesenchymal stem cells can be cultured separately^3^. Using the co-culture system, we herein analyzed the common humoral factors that changed.

Analysis of humoral factors mediating the interrelation between tumor cells and fibroblasts within the tumor microenvironment has been reported for several carcinomas such as breast cancer^18^, prostate cancer^19^, and lung cancer. There have been many studies on the relationship with normal cells in epithelial tumors, in cancer-related fibroblasts where fibroblasts acquire the properties of mesenchymal cells^20–22^. In addition, the number of studies described the effects of humoral factors on immune cells. However, the role of specific chemokines in the microenvironment of osteosarcomas was not reported yet.

We used a microarray method to provide an overview of conditions under which osteosarcoma cells are adjacent to normal cells and examined the changes that occur between these cells. MG63 cells had a higher expression level of CCL26 than hMSCs under single culture conditions; however, the expression of CCL26 was even enhanced when the tumor cells were co-cultured with the normal cells. Thus, it was found that CCL26 mutually increased expression by involving the normal cells. Research has shown that the expression of CCL26 increases when normal and malignant tumor cells share the same environment^4,23^. Given that CCL26 plays an essential role in inflammatory cell infiltration, it is suspected that it promotes tumor cell infiltration and distant metastasis. In addition, malignant tumor cells may exploit normal cells so that they contribute to tumor progression.

It is assumed that CCL26, the expression of which increases when osteosarcoma and mesenchymal stem cells are coexistent, acts in a tumor-promoting manner. When recombinant CCL26 was added as a ligand, the expression level of CCL26 increased in the same way as observed under co-culture conditions. In contrast, the expression of CCL26 decreased when neutralizing antibodies against CCL26 were added. Furthermore, the expression of CCL26 was also reduced in single culture upon the addition of the neutralizing antibodies, indicating that it also exhibits an autocrine mechanism. Thus, it was concluded that when the osteosarcoma cells and hMSCs were located close to each other, the cells mutually formed a signal loop by establishing a paracrine mechanism in addition to an autocrine mechanism. MG63 and hMSCs that were co-cultured and treated with ligands, and thus showed an elevated CCL26 expression, demonstarated a significantly increased and dose-dependent cell growth. Therefore, it was supposed that the chemokine CCL26 was not only an inflammatory substance in osteosarcoma cells but also a very important humoral factor that promotes tumor progression. CCL26 has previously been shown to increase the invasive ability of prostate cancer^4^ and hepatocellular carcinoma cells^23^, which is consistent with our results.

A more important effect of chemokine CCL26 on the microenvironment of tumor cells is the enhancement of the motility and invasive ability. Both the migratory and invasive abilities of osteosarcoma cells were enhanced by co-culturing with hMSCs accompanied by the addition of recombinant CCL26. In contrast, both abilities were significantly decreased in the group containing the neutralizing antibodies against CCL26. We also verified the mechanism by which CCL26 affects motility. CCL26 is a ligand of the CCR3 receptor that controls various signals, including Rac, Src, FAK, PI3K, and ERK^9–12^. Normally, CCL26 plays a role in eosinophil migration during allergies, but we speculated that it might also be involved in the cell migration and motility of cancer cells^24,25^. Our research focused in particular on Rac and Src and their associated pathways because they represent factors associated with the motility downstream of CCR3. As expected, we confirmed that the enhancement of CCL26 resulted in the activation of signaling involved in the invasion of tumor cells and phosphorylation of proteins associated with the proliferative capacity, such as ERK. Thus, we suggest that the cell-to-cell interaction of CCL26 enhanced not only proliferation but also motility of osteosarcoma cells. At the same time, morphological changes of the cells also revealed the effect of CCL26. There was a clear difference in actin fiber development between cells with enhanced CCL26 expression and those treated with neutralizing antibodies. This showed that intracellular signals triggered by CCL26 resulted in morphological changes.

Distant metastasis is the most severe and common type of malignancy. Complications associated with distant metastases have to be resolved to prolong the survival time of patients with osteosarcoma. We confirmed that the intraperitoneal administration of neutralizing anti-CCL26 antibodies reduced the volume of metastatic lesions in nude mice. This indicates that osteosarcoma cells could increase CCL26 in co-operation with the surrounding normal cells to enhance their invasive potential for distant metastasis formation. Furthermore, the expression of CCL26 was significantly reduced in mice tumor tissue in which CCL26 was neutralized, and thus the results observed ex vivo were confirmed in vivo. The humoral factor CCL26 does not come into direct contact with the tumor cells, but it is intimately involved in the development of distant lesions. This may lead to developments new strategy for controlling metastasis, the most lethal attribute of osteosarcoma.

For more than 40 years, there have been no new drugs for the treatment of osteosarcoma, and the current standard anticancer drugs used for osteosarcoma were developed in the 1970s. Improvement of therapeutic outcomes by combining existing anticancer drugs^26^, modifying the administration timing, and adding different drugs, has reached its limit. A completely new treatment strategy has to be developed to improve the outcomes in patients with osteosarcoma. How do osteosarcoma cells interact with the surrounding microenvironment and distant metastases? This study revealed one possible answer to this question. Drugs that directly kill tumor cells also cause significant damage to normal cells, but the focus of this studies an inflammation-specific chemokine, CCL26, usually triggered by eosinophils. Although CCL26 is a necessary factor for maintaining homeostasis, if it is also involved in promoting the development of malignant tumors via normal cells, controlling CCL26 may also lead to suppression of osteosarcoma progression. We unveiled the ability of osteosarcoma cells to use CCL26 as a factor to utilize normal cells for their growth, invasion and metastasis. This finding improves our understanding of the ecology of osteosarcoma and suggests a potential new therapeutic strategy.

## Materials and methods

### Ethical approval

Each author certifies that his or her institution has approved the animal protocol for this investigation and that all investigations were conducted in conformity with ethical principles of research. Mouse experiments were approved by the Medical Ethics Committee of Oita University (No. 182403) and all experiments were performed in accordance with relevant guidelines and regulations. All animal experimental procedures were performed in accordance with ARRIVE guidelines^27^.

### Cell lines

The human osteosarcoma cell line MG63 was obtained from RIKEN Cell Bank (Tsukuba, Japan). hMSCs were purchased from TaKaRa Biotechnology (Otsu, Japan). Each line was authenticated as to genotype and phenotype by the source company. MG63 and hMSCs were maintained as described previously^28^.

### Co-cultured condition

hMSCs and MG63 cells were seeded at 1 × 10^5^ cells/well individually during polymerization of the collagen type I lattice and cultured on opposite sides of a 1-µm pore, six-well cell culture insert (Becton Dickinson, Sparks, MD, USA). Cells were incubated for 48 h at 37 °C and 5% CO_2_, and total RNA was isolated from two inserts for each cell type as described previously^3^. All experiments were performed in duplicate.

### RNA isolation

mRNAs were prepared from the triplicated cell cultures using RNeasy kit (Qiagen, Valencia, CA, USA) according to the manufacturer’s instructions. The RNA quality was ensured, before labeling, using an RNA 6000 Nano kit and a Bioanalyzer 2100 (Agilent, Santa Clara, CA, USA) as described previously^28^.

### Analysis of mRNA expression by cDNA arrays

GeneChip Genome HG U133 Plus 2.0 Array (Affymetrix) was used for mRNA expression profiling in MG63, HOS, Saos, NY and MRC5 as described previously^29^. Analysis of variance was used to determine those probe sets that were significantly different between the two groups. The gene list was filtered with a fold-change cutoff of 2, resulting in the output of a list with genes that had significant differential expression at twofold or greater differences.

### Recombinant CCL26 administration

Recombinant human CCL26 (R&D system, USA) (10 ng/ml) was administered to the culture medium. After 48 h of incubation following the administration, the cells were harvested and processed for further analysis. The experiment was repeated three times.

### Neutralization of CCL26 function using anti-CCL26 antibody

Neutralizing monoclonal antibody targeting CCL26 (anti-CCL26 Ab) was purchased from Invitorogen (#MA5-23858). The cell lines were harvested 48 h after the administration of CCL26 and anti-CCL26 Ab (100 ng/ml), then subjected to various analyses. The experiment was repeated three times.

### Cell proliferation assay

The cells were plated in 6-well plates (1 × 10^5^ cells per well), and were treated with or without rCCL26 and neutralizing anti-CCL26 Ab. The concentrations of rCCL26 added to MG63 cells were 0, 0.1, 1, 10, 100 ng/ml. After 48 h of cultivation, the cells were counted using a TC10 Automated Cell Counter (Bio-Rad) as described previously^3^. BrdU proliferation assay kit (BrdU Cell Proliferation Kit; Merck, Japan) was used to perform cell proliferation assays according to the manufacturer’s instructions.

### Quantitative real time PCR

Total RNA was extracted from prepared cultured cells with TRIzol reagent (Invitrogen) and cDNA was synthesized according to the manufacturer's protocol (Roche). Quantitative real-time PCR (qRT-PCR) was performed using a Light Cycler 480 Probe Master System (Roche), and PCR-specific amplification was conducted using the LightCycler^®^ Nano (Roche) as described previously^28^. The relative expression of CCL26 and glyceraldehyde-3-phosphate dehydrogenase (GAPDH) was calculated using the 2-(ΔΔCt) method method. The primers and probe kits ofCCL26 and GAPDH were obtained from Applied Biosystems (Nagoya, Japan).

### Western blot

The details of Western blot analysis were referred to previous publication^30^. After cutting the membrane in each molecular weight, we reacted primary antibodies. Antibody produced in rabbits for CCL26 was purchased from Invitrogen (#MA5-23858) and GAPDH (#5174) was purchased from Cell Signaling Technology (Tokyo, Japan). All primary antibodies were used at a 1:1000 dilution. Peroxidase-conjugated anti-Rabbit IgG secondary antibodies (GE Healthcare) were used at a 1:2000 dilution. Three independent experiments were performed for each analysis, and the same experimental conditions were used for all gels.

### Cell motility and migration assays

Cell motility assay was carried out as previously described^31^. The changes in the expression of motility related proteins were analyzed by western blot analysis using primary antibodies against Rac1/2/3 (#2465), RhoA (#2117), Cdc42 (#2466), Src (#2109), p-Src (#2101), FAK (#3285), p-FAK (#8556), ERK (#4695), p-ERK (#4370) were purchased from Cell Signaling Technology (Tokyo, Japan). The experiments were carried out in triplicate.

### Immunofluorescence analysis

Immunohistochemistry was used to measure the levels of Rac and p-Src in the cells. After PBS washing, rehydrated culture dishes were incubated with primary antibodies against Rac (ab155938) and p-Src (ab40660) purchased from Abcam (Tokyo, Japan) diluted at 1:100 in Ab Diluent (Dako ChemMate; Dako, Japan) overnight at room temperature. For staining with Alexa Fluor 488 anti-rabbit IgG (Invitrogen, Carlsbad, CA, USA), secondary antibodies were diluted at 1:200 in Ab Diluent and added for 60 min at room temperature in the dark. Digital images were taken on a BIOREVO microscope equipped with a confocal microscopy system (BZ-9000, Keyence, Japan) as described previously^28^.

### In vivo tumor-bearing nude mouse model

The experimental metastasis model was established by injection of 1 × 10^6^ cells suspended in 100 μl of normal saline into the tail veins of nude mice as described previously^3^. Five groups were generated: (1) untreated MG63 cells (MG63) (n = 5); (2) MG63 cells treated with rCCL26 (MG63 + rCCL26) (n = 5); (3) MG63 cells co-cultured with hMSCs (MG63 + hMSCs) (n = 5); (4) MG63 cells co-cultured with hMSCs and treated by anti-CCL26 Ab (MG63 + hMSCs + anti-CCL26 Ab) (n = 5); and (5) MG63 cells treated with antiCCL26 Ab (MG63 + anti-CCL26 Ab) (n = 5). All mice were fed in standard conditions with weight monitoring and sacrificed 6 weeks after the cell inoculation. All mice used in this study were anesthetized with ketamine/xylazine or isoflurane/oxygen for experiments. Tumor volumes were measured using a micro-CT apparatus which allows us to obtain high-resolution CT images in small living animals. The tumor volume of the lung nodule was estimated using the formula: (π × long axis × short axis × short axis)/6. The resected tumors were fixed with 4% formaldehyde, paraffin embedded, sectioned using microtome, and reacted with Rac (ab155938) was purchased from Abcam (Tokyo, Japan) and p-Src (#2105) antibodies was purchased from Cell Signaling Technology (Tokyo, Japan). The expression of proteins in the section was visualized using DAB and EnVision System (Dako) (Supplementary Information).

### Statistical analysis

A two-tailed Student’s t-test was used for the analysis of continuous variables. We determined the differences among more than three groups using a non-repeated measure analysis of variance (ANOVA) and Scheffe test. Results were expressed as the mean ± standard deviation, and p < 0.05 was considered as statistically significant. All statistical analyses were done using SPSS 24.0 software (IBM, Tokyo, Japan) like previous description^32^.

## Supplementary Information

Supplementary Information.
