# Supplementary material for: Analysis of the signal cross talk via CCL26 in the tumor microenvironment in osteosarcoma
Source: Sci Rep. 2021 Sep 13;11:18099. doi: 10.1038/s41598-021-97153-2 (PMC8438066; doi:10.1038/s41598-021-97153-2)
Supplement: Supplementary file 1 — Supplementary Information. [file 41598_2021_97153_MOESM1_ESM.pdf]

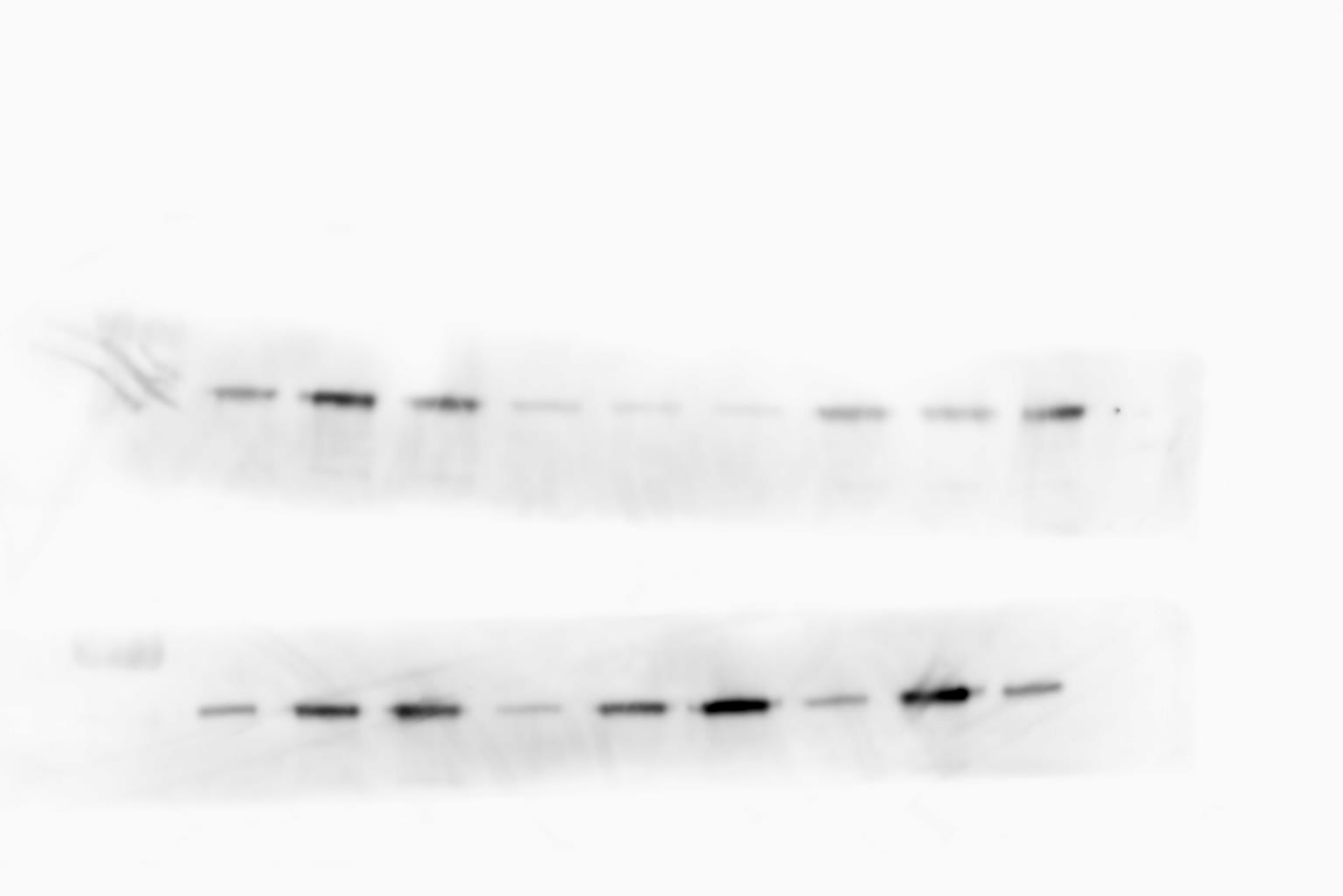

Supplementary Figure S1. The full length of the Western blot used in this study is shown.
We evaluated protein expression analysis by Western blot. After blocking, the membrane is cut according to the molecular weight and then reacted with the primary antibody.

Fig.2. (E) CCL26

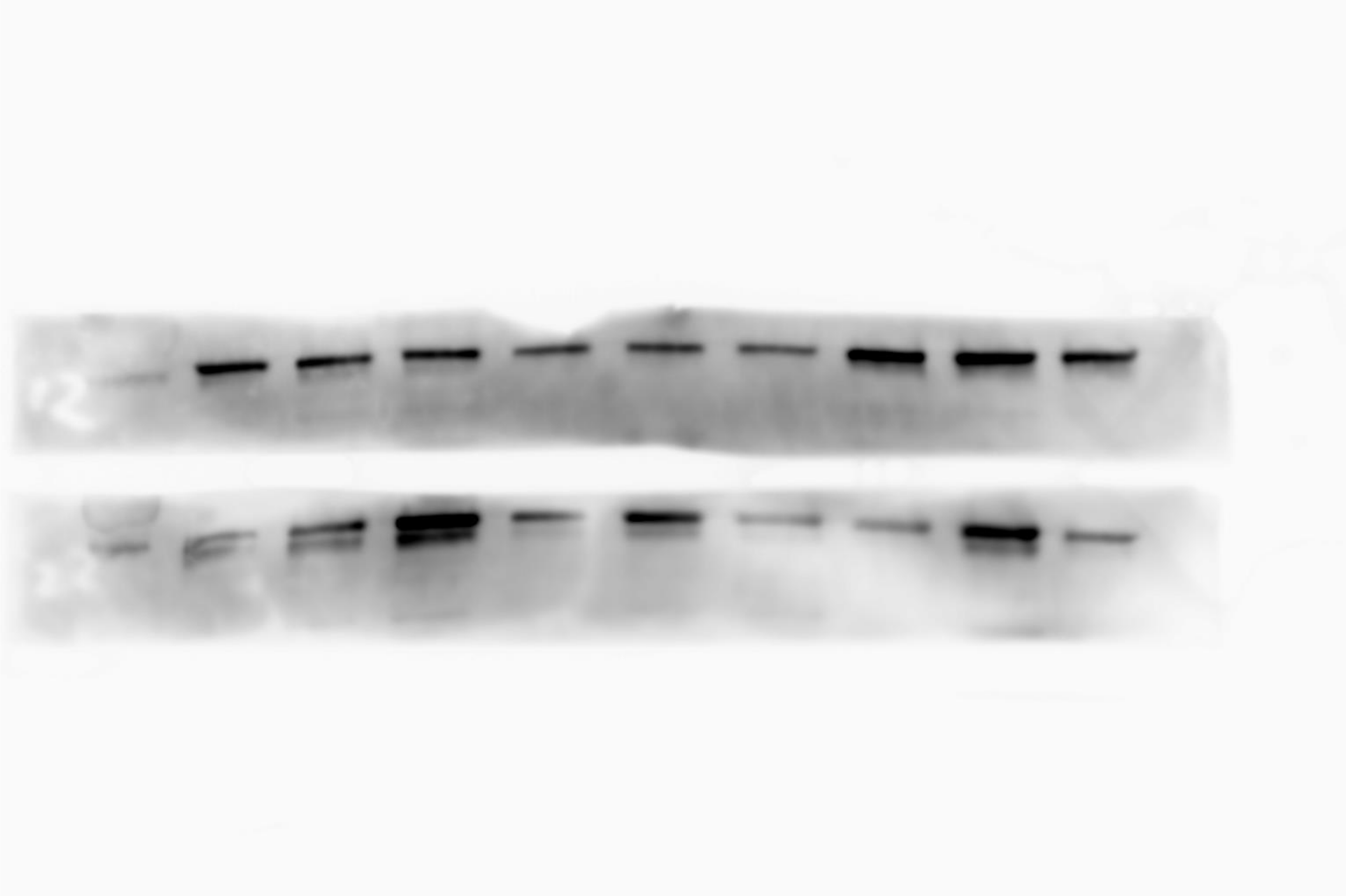

Fig.2. (E) GAPDH

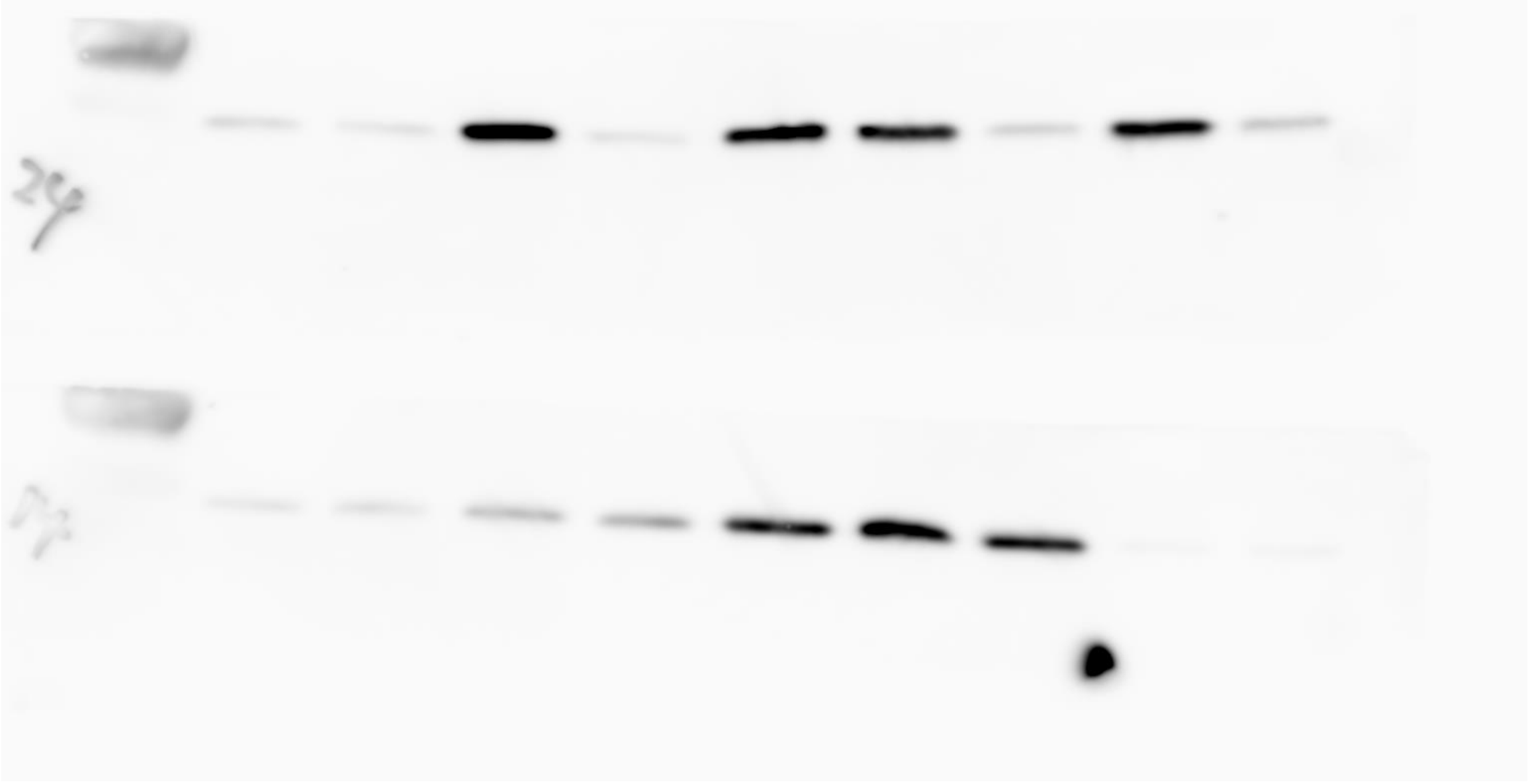

Fig.3. (C) CCL26

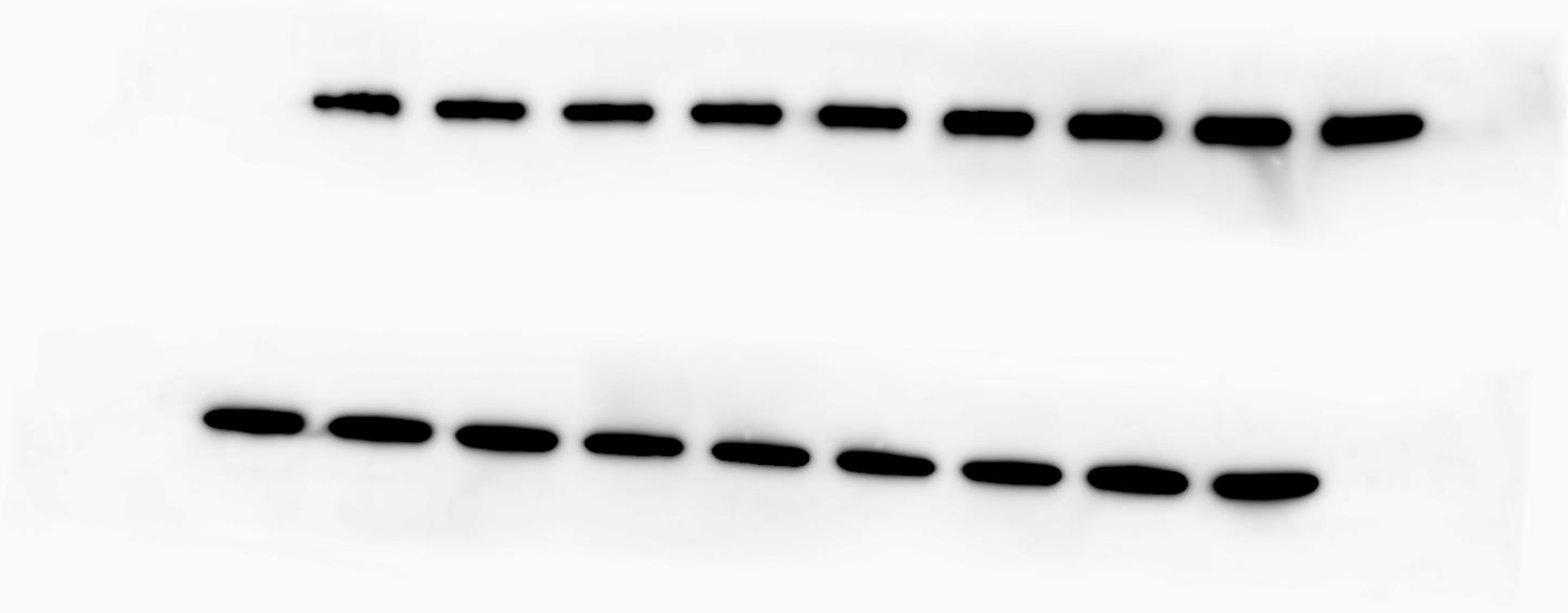

Fig.3. (C) GAPDH

Fig.5. (A) GAPDH

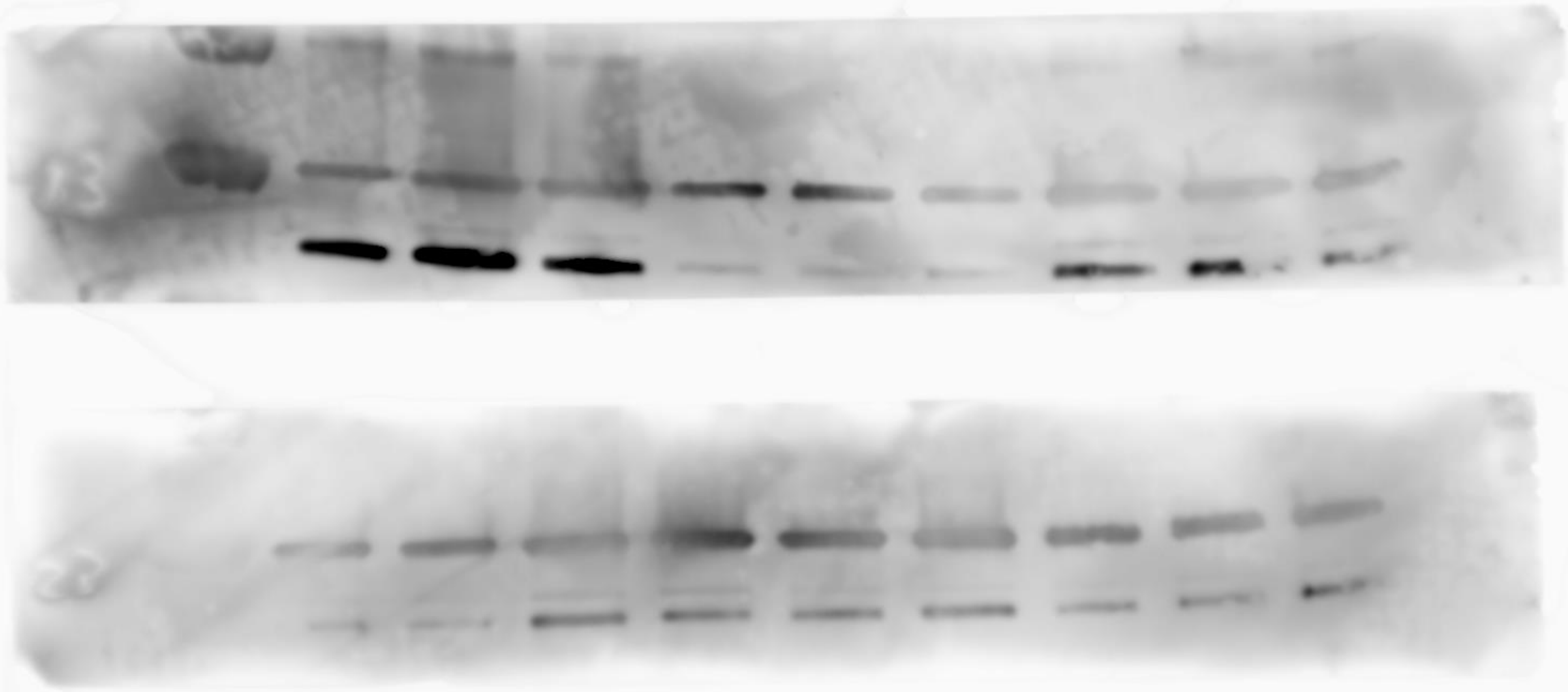

Fig.4. (A) Rac

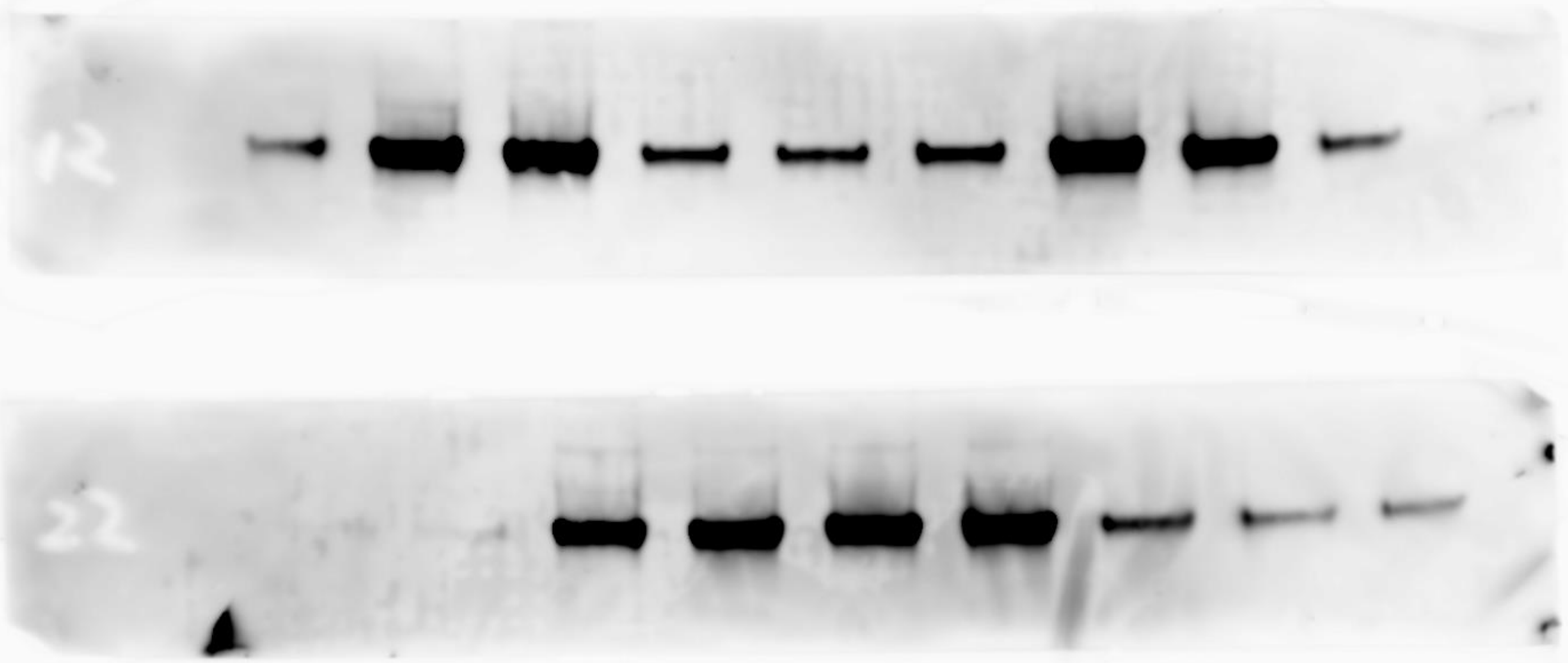

Fig.4. (A) RhoA

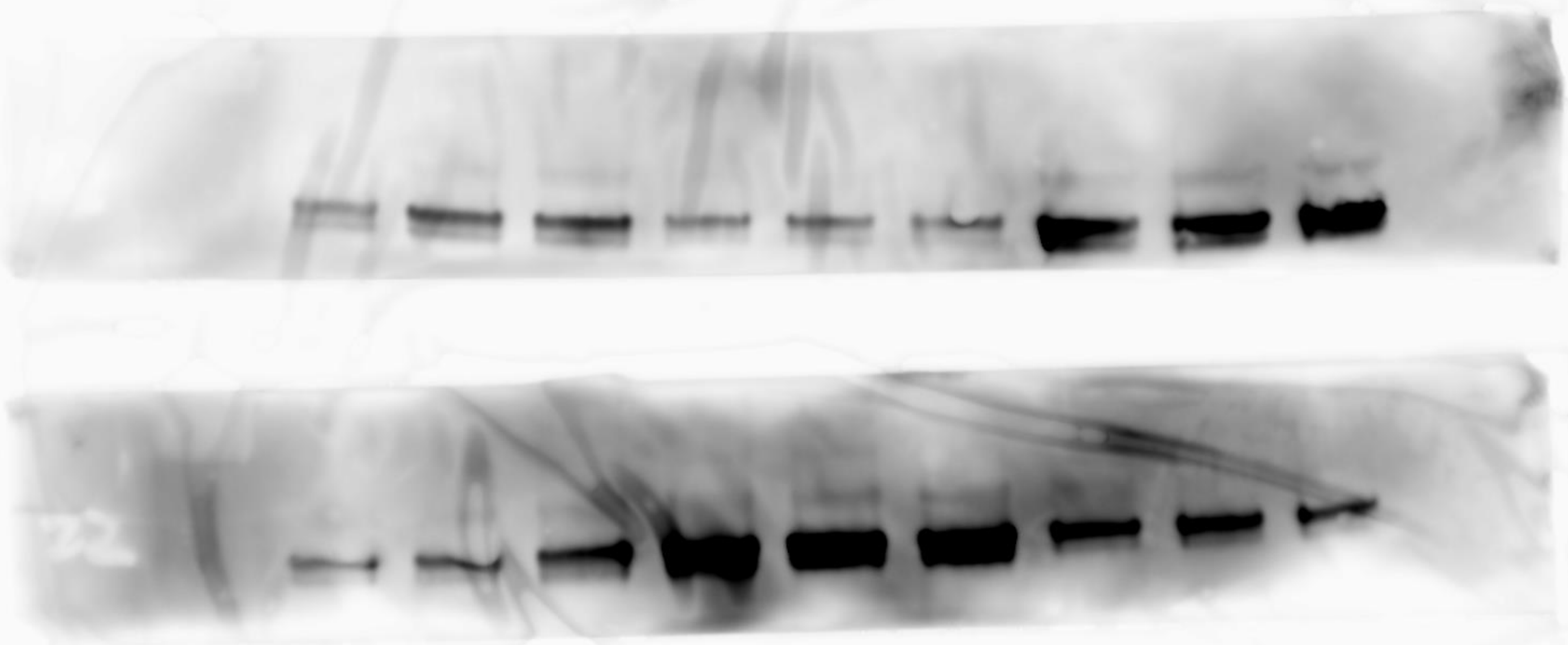

Fig.4. (A) Cdc42

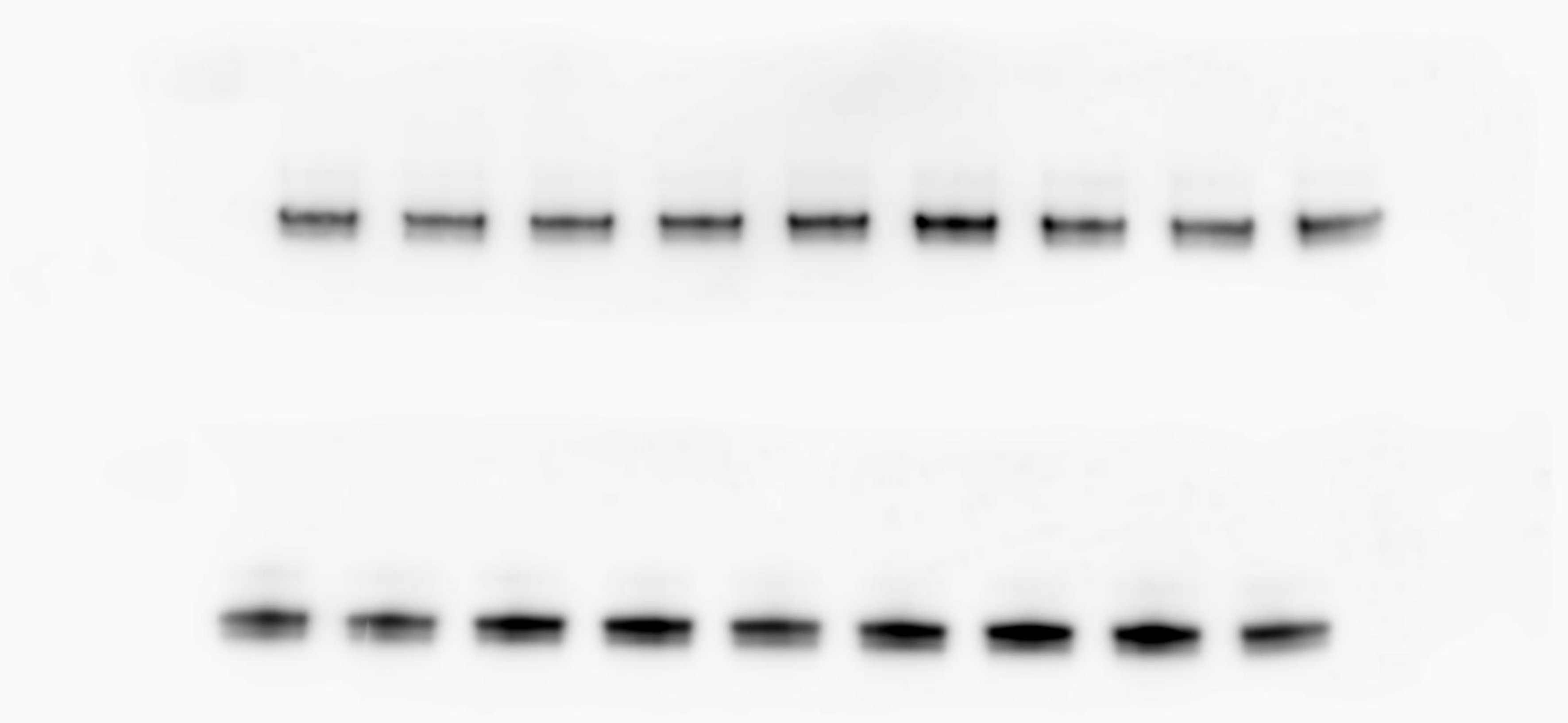

Fig.4. (A) GAPDH

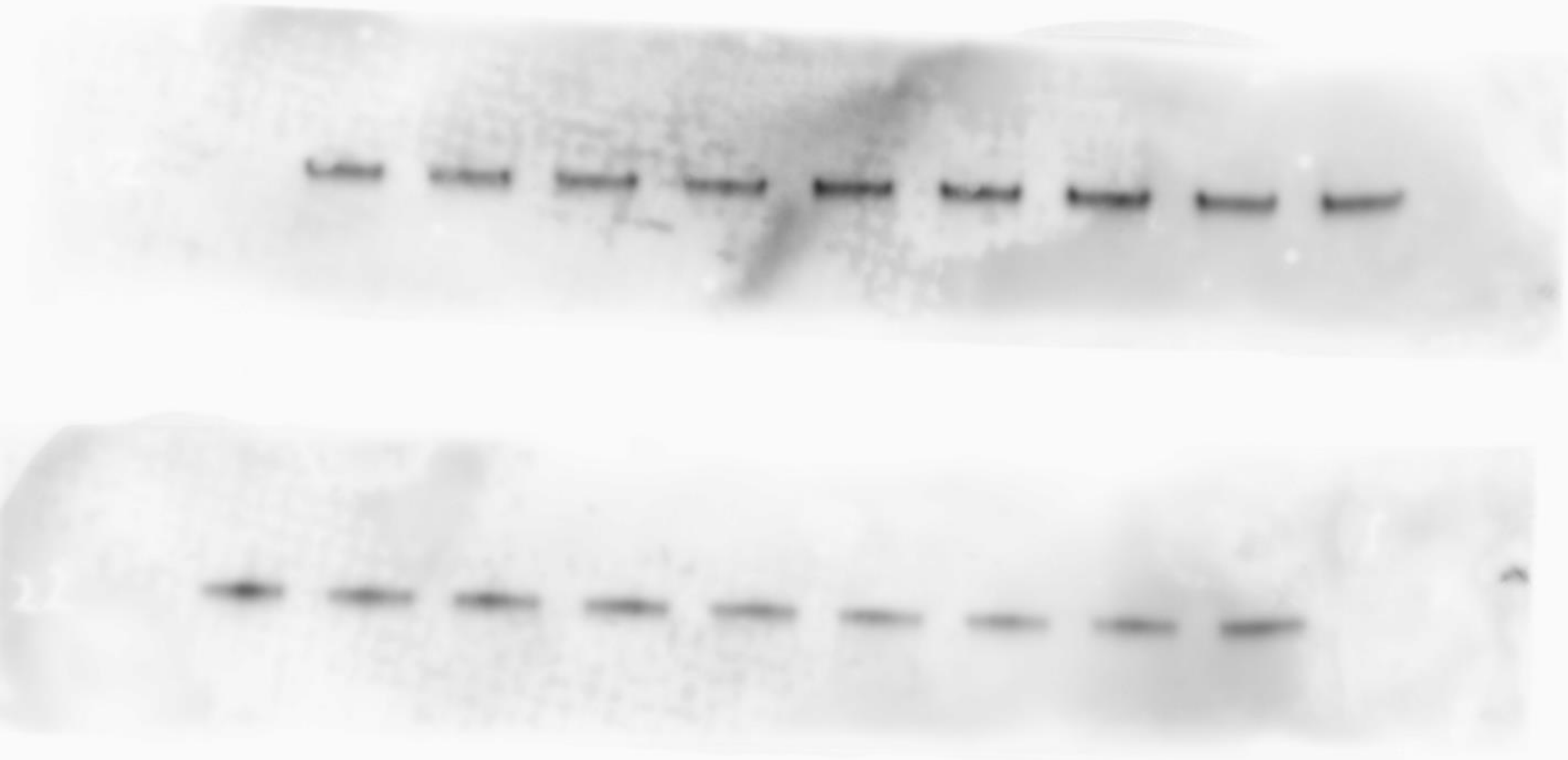

Fig.5. (A) Src

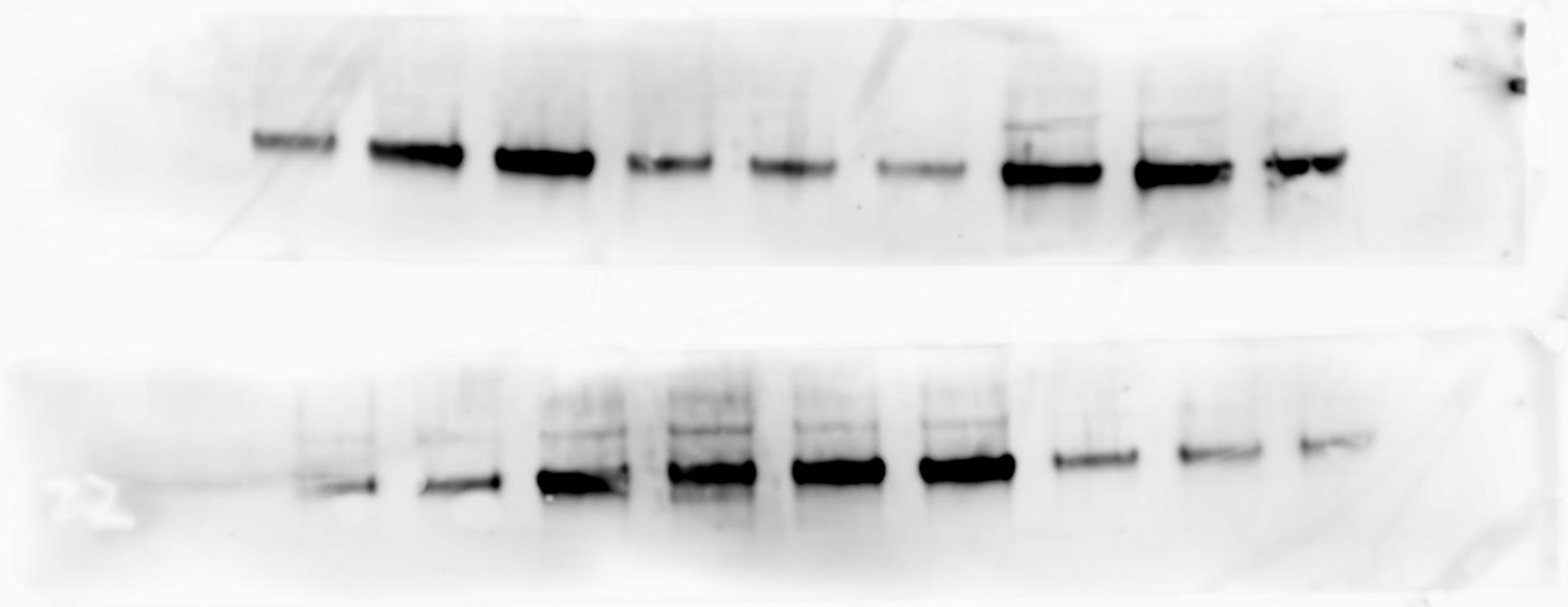

Fig.5. (A) p-Src

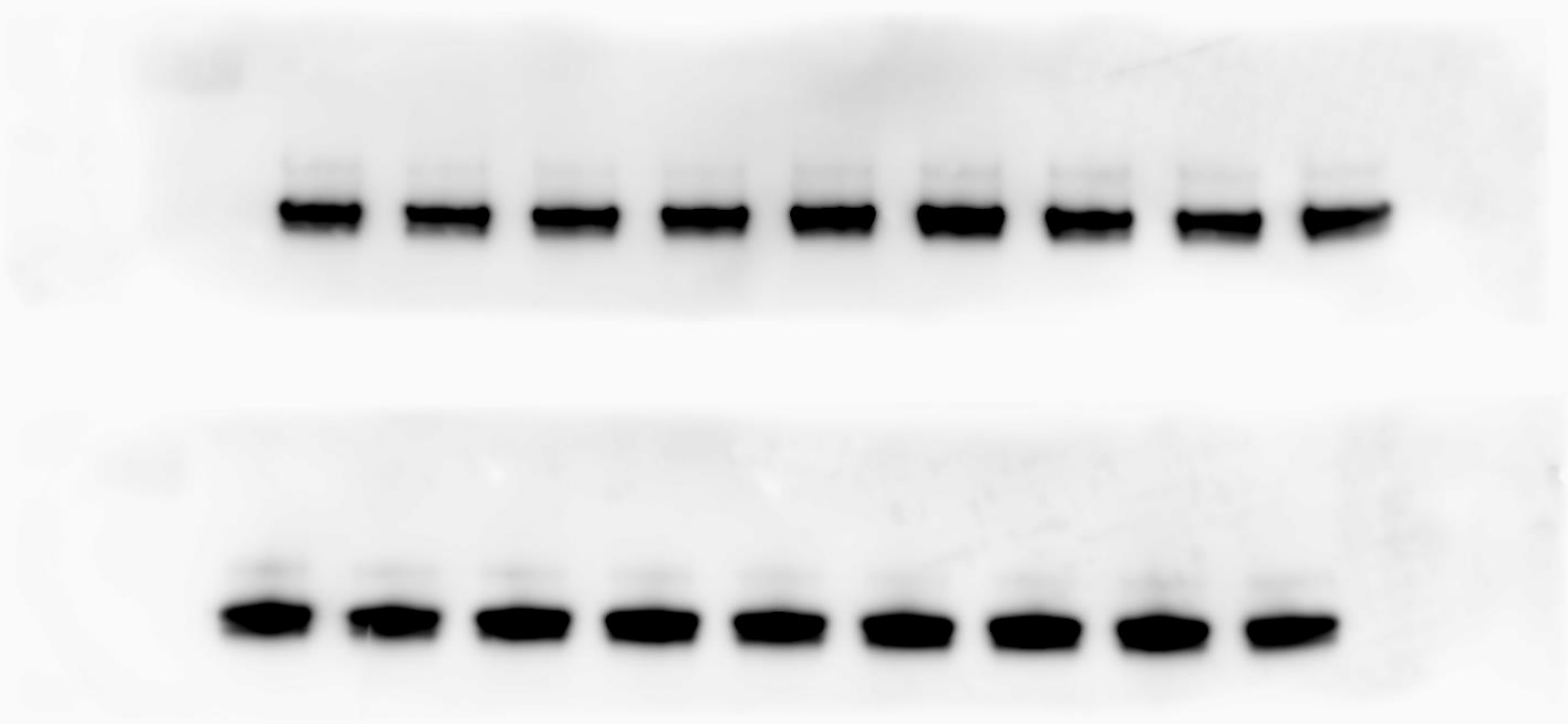

Fig.5. (A) FAK

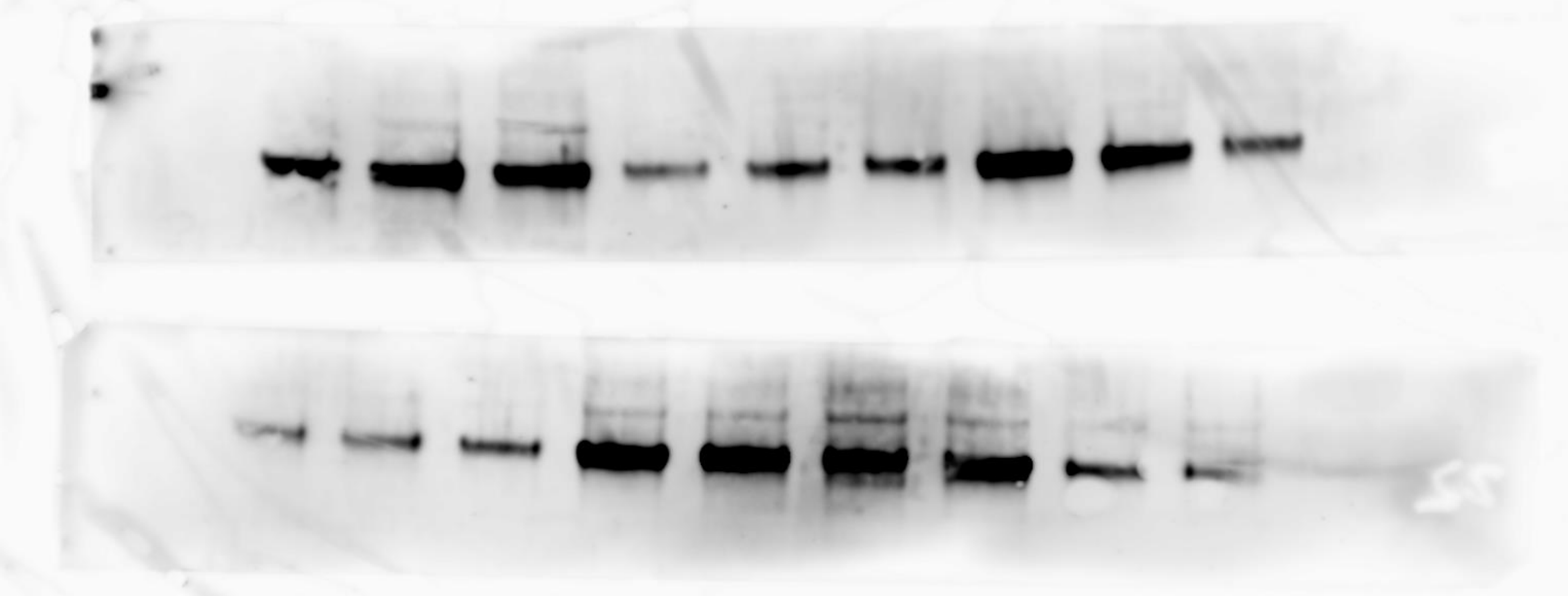

Fig.5. (A) p-FAK

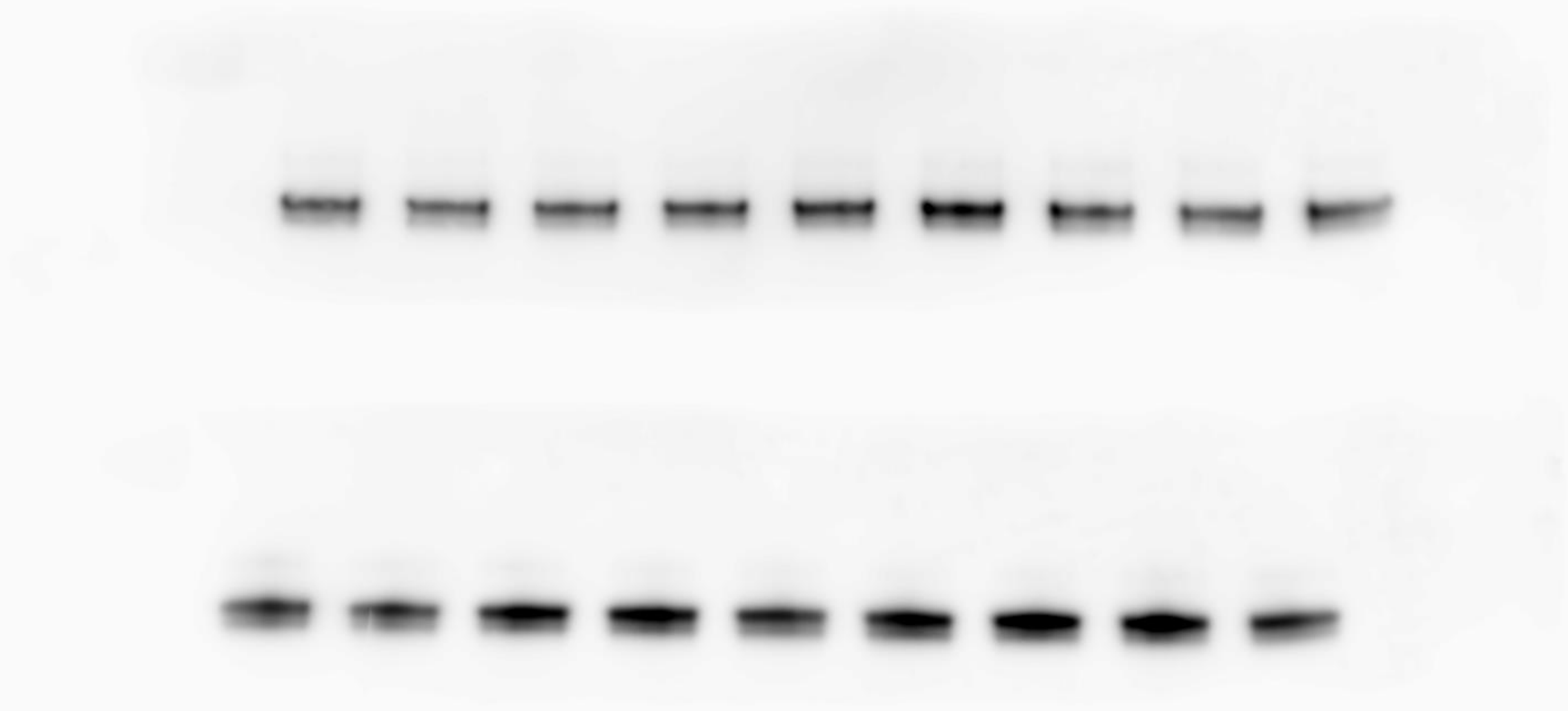

Fig.5. (A) MEK

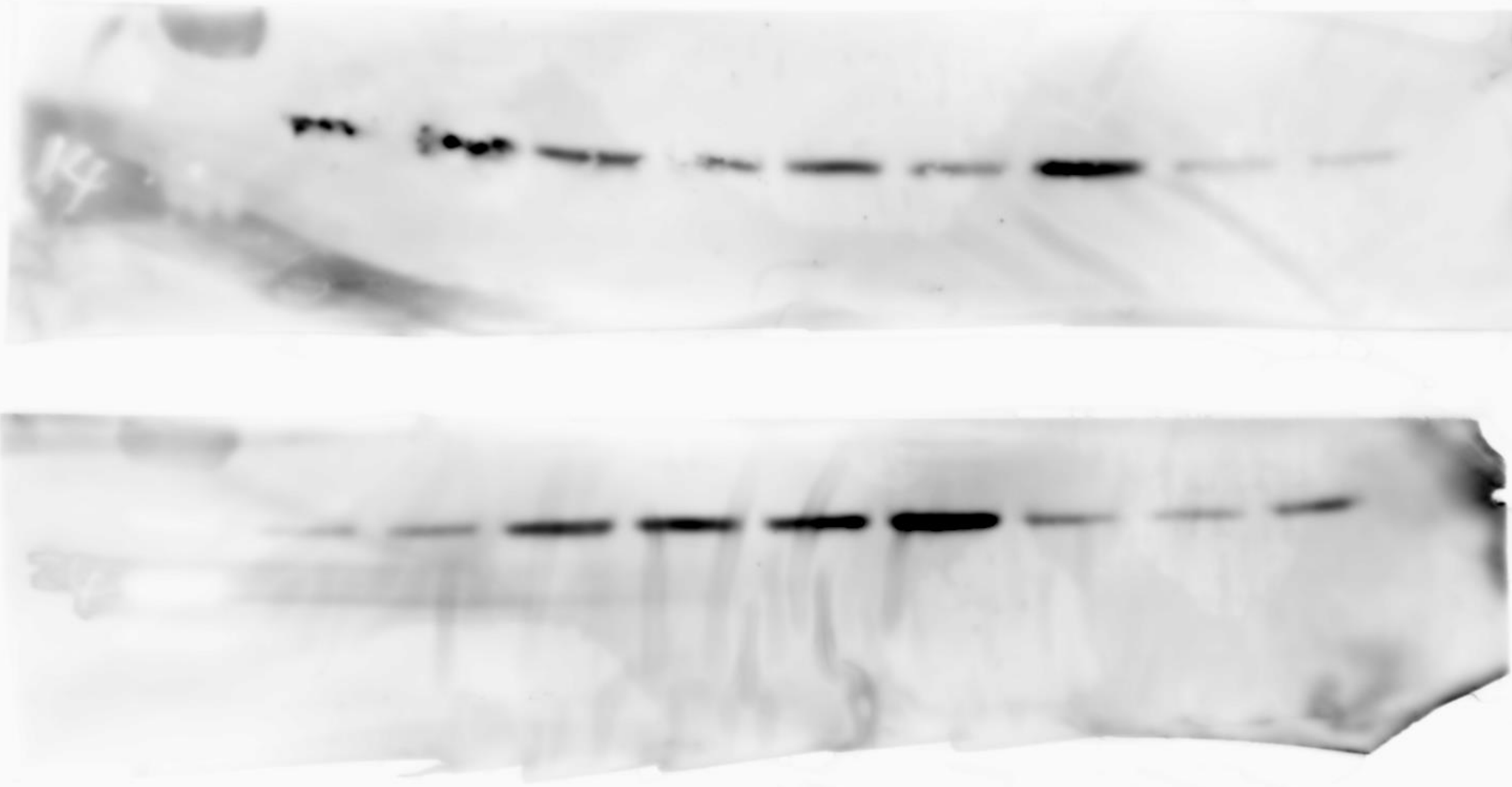

Fig.5. (A) p-MEK

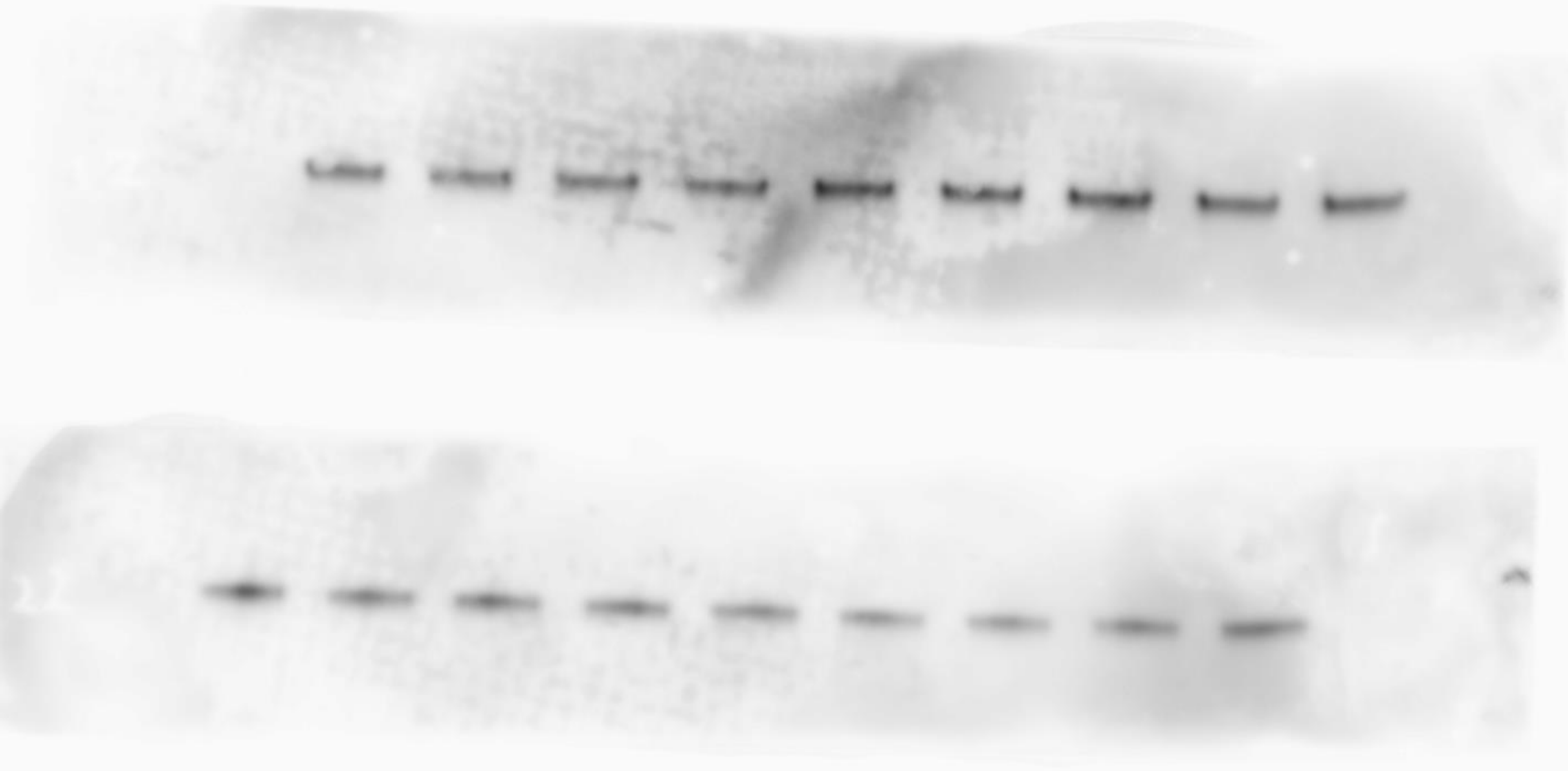

Fig.5. (A) ERK

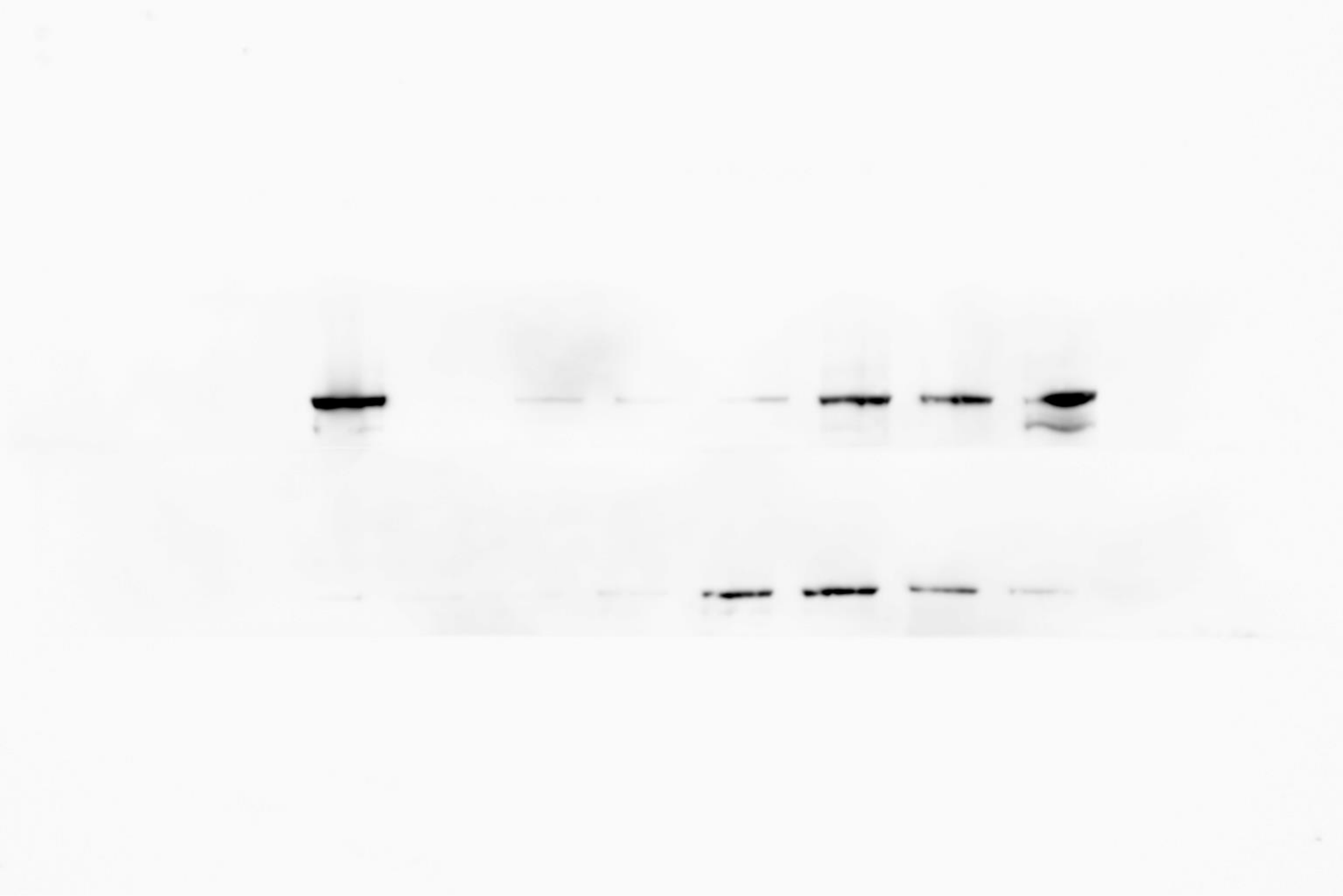

Fig.5. (A) p-ERK
